# 2D Materials in Development of Electrochemical Point-of-Care Cancer Screening Devices

**DOI:** 10.3390/mi10100662

**Published:** 2019-09-30

**Authors:** Mohsen Mohammadniaei, Huynh Vu Nguyen, My Van Tieu, Min-Ho Lee

**Affiliations:** School of Integrative Engineering, Chung-Ang University, Heukseok-dong, Dongjak-gu, Seoul 06910, Korea; mniaei@gmail.com (M.M.); huynhvu0205@gmail.com (H.V.N.); tmvantp0113@gmail.com (M.V.T.)

**Keywords:** point-of-care, cancer, diagnostics, biosensor, electrochemical, 2D material, graphene, MoS_2_, Bi_2_Se_3_, MXene

## Abstract

Effective cancer treatment requires early detection and monitoring the development progress in a simple and affordable manner. Point-of care (POC) screening can provide a portable and inexpensive tool for the end-users to conveniently operate test and screen their health conditions without the necessity of special skills. Electrochemical methods hold great potential for clinical analysis of variety of chemicals and substances as well as cancer biomarkers due to their low cost, high sensitivity, multiplex detection ability, and miniaturization aptitude. Advances in two-dimensional (2D) material-based electrochemical biosensors/sensors are accelerating the performance of conventional devices toward more practical approaches. Here, recent trends in the development of 2D material-based electrochemical biosensors/sensors, as the next generation of POC cancer screening tools, are summarized. Three cancer biomarker categories, including proteins, nucleic acids, and some small molecules, will be considered. Various 2D materials will be introduced and their biomedical applications and electrochemical properties will be given. The role of 2D materials in improving the performance of electrochemical sensing mechanisms as well as the pros and cons of current sensors as the prospective devices for POC screening will be emphasized. Finally, the future scopes of implementing 2D materials in electrochemical POC cancer diagnostics for the clinical translation will be discussed.

## 1. Introduction

Cancer is expected to be the first leading cause of death worldwide in the 21st century [1]. Therefore, the early detection and point-of-care (POC) diagnosis of cancer is vital for increasing the chance of successful treatments and overall survival [2]. Developing a simple (POC) screening device with high utility, acceptability, and cost effectiveness that does not involve the need for experts and sophisticated equipment would be highly demanding, specifically when the test is carried out in the socially-disadvantaged and resource-limited areas [3]. In particular, cancer diagnosis is on the basis of biomarker detection. Cancer biomarkers are mainly the chemical substances, including nucleic acids (mutated DNA/RNA, mRNA, or short strand microRNAs), tumor associated antigens, secreted proteins, and small molecules [4,5]. These biomarkers are typically produced during the tumor progression when their expression levels change. However, in most of the cases the patient with cancer develops symptoms when it is quite late for an effective treatment [6]. As a result, cancer diagnosis at a very early stage of tumor promotion would be highly demanding for the benefit of improving the human health and survival rate. However, some of the biomarkers are expressed at the ultra-low levels during the early stage of the diseases to make the diagnostics very challenging [7]. Another important factor would be the fact that single biomarker detection does not provide enough information for the clinicians to perform an accurate diagnosis, which results in poor prognosis [8]. Therefore, the fabrication of a very sensitive device for detecting and profiling multiple biomarkers at the onset of tumor promotion is in a great demand. 

**Figure 1:**
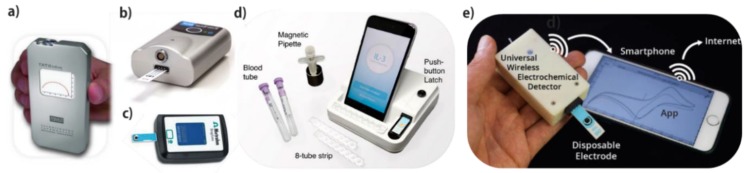
Examples of hand-held electrochemical readers as (**a**) PocketStat (IVIUM Technologies^®^), (**b**) EmStat (PalmSens^®^) and (**c**) DropStat (DropSens^®^); (**d**) integrated biosensor for Sepsis diagnosis (adapted from reference [13] with permission of American Chemical Society); and, (**e**) universal wireless electrochemical detector (UWED). Reproduced with permission from [14].

Recent advances have been the use of colorimetric paper-based [9] and lateral flow assays (LFAs) [10] as well as electrochemical apparatuses [11] to demonstrate the most promising POC diagnostics and screening methods. Amongst them, electrochemical devices with the advantages of low cost, high sensitivity, miniaturization capability, requirement of low sample volume, and low power supply have represented very good candidates for the POC screening, since the end-users always request for simple, fast, and affordable technologies (Figure 1) [12]. However the fabrication of a qualified POC device for cancer screening is still an ongoing challenge and it necessitates interdisciplinary research and development to achieve a product that meets all of the requirements for the POC cancer screening.

In this review, electrochemical sensing systems will be discussed and their applications in quantification of a number of cancer biomarkers, including oncogene-related nucleic acids (DNA or RNA), proteins (carbohydrate antigen, carcinoembryonic antigen, enzymatic tumor markers, etc.), and small molecules (reactive oxygen species (ROS) and reactive nitrogen species (RNS)), will be considered. However, the main focus of this review is the role of sensor transducer on the performance of the biosensor, particularly the engagement of two-dimensional (2D) materials in improving the sensitivity and efficiency of electrochemical POC devices will be summarized and compared. Some newly-emerged 2D materials will be also introduced and their applications in POC screening will be also covered. Finally, the pros and cons of different methods as well as the challenges faced by electrochemical biosensors in translation to the clinical approaches will be given.

## 2. POC Screening

POC screening or bedside testing refers to rapid medical diagnostic testing that is carried out near the patient [15]. Conventional methods that are performed in medical laboratories require a high volume of specimens (e.g., blood) in which the analysis takes hours to days. These methods are either time-consuming and laborious or costly and skill-oriented, which makes them ineffective when there is a need for continuous disease monitoring and management, specifically in non-developed and developing countries with limited resources [16]. Various prospective sensing techniques are being used for POC screening, however optical and electrochemical methods are studied to be more appropriate. The integration of these methods with technologies, such as microfluidics, paper strips, and smartphone readouts, have paved the way to develop desired automated, portable, and easy-to-use POC screening devices [17,18,19].

Smartphones have offered very useful readout systems due to their accessibility and handiness to realize the POC digital health monitoring. Smartphones can record data using either the optical camera or a data transfer interface via the lightning connector and simply convert the obtained information to meaningful results for quantitative analysis by a previously-designed mobile application. These data can be stored in the cloud for ease of access and further data communication with clinicians. On the other hand, to realize the device automation, microfluidics technology and LFA have been combined together to offer a new class of practical POC device of microfluidic paper-based analytical device (µPAD), which has been receiving increasing attention from various academic and industrial sectors due to its low cost, disposability, and biocompatibility [17]. Unlike the microfluidic devices, µPADs do not need pumps and work based on the capillary action to considerably reduce the cost, size, and complexity of the device as one of the most successful commercial µPADs is the pregnancy test. However, the major concern that is faced by µPADs would be their sensitivity, since the detection mechanism is mainly based on the colorimetric approaches which makes them unable to detect some cancer biomarkers with ultra-low abundance of aM ~ fM (e.g., microRNAs). Various POC devices have been developed and some of them are commercially available, which will be discussed in this review.

## 3. Electrochemical Biosensors

**Figure 2:**
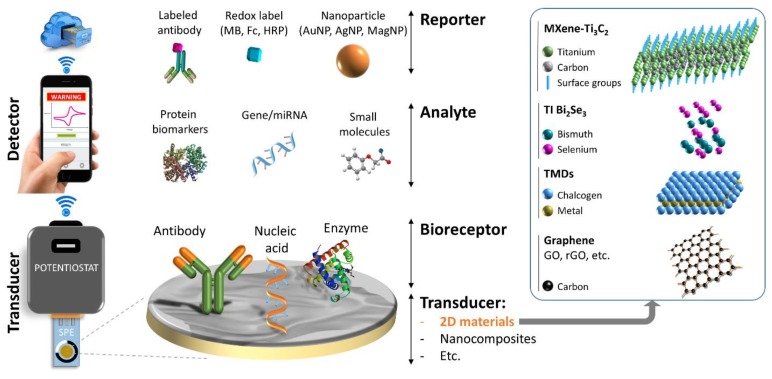
Schematic representation of an ideal electrochemical point-of-care (POC) screening device composed of a disposable screen printed electrode (SPE), a portable wireless potentiostat, a smart phone detector and further cloud storage. General configuration of different segments of electrochemical detection system is illustrated with the focus on four types of two-dimensional (2D) materials (graphene and its derivatives, transition metal dichalcogenides (TMDs), topological insulator (TI) Bi_2_Se_3_, and MXene-Ti_3_C_2_).

Figure 2 demonstrates a schematic design of an electrochemical device. According to the International Union of Pure and Applied Chemistry (IUPAC), an electrochemical biosensor is an integrated apparatus that is composed of a “bioreceptor” in an intimate contact with a “transducer” element that is designed to provide semi- or fully-quantitative analysis of the target analyte [12]. However, “detector”, as the third element, plays an important role in the development of POC devices. A bioreceptor is composed of biological recognition materials (antibody, nucleic acid, enzyme, polymer, microorganism, etc.) to function as the analyte recognition elements. Transducer includes two parts of (**i**) solid electrode that is composed of conductive materials (gold, silver, platinum, 2D materials, etc.) from which the electrochemical reaction occurs in the presence of electroactive substances (redox reporters), and (ii) signal processor (potentiostat) that is an electronic hardware to control and run the electroanalytical performance of the species on a three electrode electrochemical cell and convert the transmitted signal to a detectable electrical signal. Detector (e.g., PC, Smartphone, Watch, etc.) is to read the electrical signal, analyze the output data, and convert it to meaningful measures.

To realize electrochemical POC screening, (**i**) the bioreceptor is supposed to be very selective; (ii) transducer should be versatile, reliable, sensitive, and inexpensive; (iii) signal processor is to be small, portable, and require low power; (iv) detector needs to be an easy-to-use tool; and finally, (**v**) the POC device should demonstrate a complete automation of sample analysis on a single integrated platform, allowing for the end-users to perform tests without special expertise and trainings. 

Endeavors have been made to fabricate reliable electrochemical POC devices that are based on the above-mentioned requirements by miniaturizing potentiostats, developing screen printed electrodes and utilizing smartphones for signal readout (Figure 1). Many immunosensors, genosensors, enzyme-based sensors, chemosensors, and cytosensors have been developed, although one of the great examples of electrochemical POC devices that has dominated the diagnosis market is the glucose sensor for diabetes monitoring on the basis of amperometric detection of glucose on enzyme-modified screen-printed electrodes coupled with handheld transducers [20]. In 1987, the first portable blood glucose device named ExacTech^TM^ was launched by MediSense Inc (East Coast, New England) [21]. This pen-sized digital product could effectively monitor the blood glucose level in 30 s. Its success led to the production of various devices with improved performances, favorable for POC testing by diabetic patients. These devices, which have shown high sensitivity and selectivity, low cost, simplicity, and requirement of few microliters of sample volume, have being successfully used by both patients and clinicians frequently. Abbott Laboratories MediSense Products (Bedford, MA, USA) later released Sof-Tact^TM^ as the first automated POC device that is capable of lancing, sample collection, and glucose quantification in a single step [22]. In 1999 towards multiple detection, same company commercialized Precision Xtra^TM^ for real-time monitoring of both glucose and ketone levels in blood, which could provide important information regarding the diabetes development stages. The fifth generation of strip testing technology on the basis of a three-electrode design, called MediSense TrueMeasure™ (Abbott Laboratories, Abbott Park, IL, USA), was introduced to increase the accuracy of glucose testing with high degree of selectivity to perform the precise detection of glucose within more than 60 interferences. Various commercially-available devices that were fabricated by large diagnostics companies, such as Roche Diagnostics, Bayer, Abbott, etc., aiming to reduce the sampling volume and improve the simplicity and sensing accuracy, have been thoroughly summarized in a review that was provided by J.D. Newman and A.P.F. Turner [23]. 

Recently, self-powered electrochemical devices have been introduced to remarkably advance the POC screening concept. In these devices, the required electrical power is not supplied by the external sources, but the device by itself. One example would be the self-powered paper-based electrochemical devices (SPEDs), as reported by Ramses V. Martinez for the quantitative detection of multiple targets of glucose, uric acid, and L-lactate [24]. The device consists of a screen-printed electrode on a paper-based microfluidic chip coupled to a pocket-sized rechargeable potentiostat with the ability of being powered by the user via a built-in tribogenerator. The favorable outcomes of glucometers have inspired the development of electrochemical POC devices for cancer screening based on biomarker detection.

### Electrochemical Biosensors/Sensors for Cancer Biomarker Detection

In general, cancer biomarkers can be classified into four groups of (**i**) risk screening, (ii) predictive, (iii) prognostic, and (iv) diagnostic markers [25]. Among them, diagnostic biomarkers, including particular proteins and genes, are vital for assisting the early diagnosis of cancers when the patients have not yet developed any distinctive symptoms. During the past decade, many genomic and proteomic techniques have been developed to detect abnormal gene expressions and mutations during the cancer progression. Some of the well-known methods are polymerase chain reaction (PCR) amplification [26], DNA sequencing [27], microarray [28], and northern blotting (NB) [29]. Despite their high throughput, they are costly methods and they require tedious procedures and specific equipment. Likewise, various protein detection techniques have been reported for cancer diagnostics. Some of the commercially available methods for the clinical analysis of proteins involve multiplexed enzyme-linked immunosorbent assay (ELISA) kits called Q-Plex^TM^ (Quansys Bioscience, Logan, UT, USA), electrochemiluminescence (ECL)-based technology called Cobas^®^ (Roche diagnostics, Rotkreuz, Switzerland), fluorescence-based technology (Luminex’s xMAP, Luminex Corporation, Austin, TX, USA), and surface plasmon resonance (SPR)-based technique, called ProteOn^TM^ XPR36 (BioRAD, Hercules, CA, USA). Besides their high efficiency, transforming these techniques to portable and cost-effective technologies that are suitable for POC applications demand more technical efforts and dedications. 

On the other hand, electrochemical detection methods are being progressively studied to be the prospective strategies for POC cancer screening applications [30]. Although, despite the impressive improvements in the cancer biomarker detection, there still exist many challenges to address. One of the main challenges would be the fabrication of a very robust and sensitive platform for the effective detection of cancer biomarkers with a low limit of detection and wide dynamic detection range in clinical samples (body fluids: blood, serum, saliva, urine, etc.). 

Remarkable 2D material-based electrochemical biosensors/sensors for cancer diagnosis have been reported with improved sensitivity and even selectivity with the emergence of 2D materials and integration with sensing devices [31]. 2D materials can be incorporated with the electrochemical biosensors as signal enhancers either in electrode modification or in sandwich assays. Their enzyme-like behavior can be also used for the development of very sensitive chemosensors [32]. These functional materials not only can provide high conductivity and sound catalytic behavior, but also assist with the target recognition via specifically-designed signal reporters. Several 2D materials in the form of nanoparticle, nanosheet, nanorode, nanotube, nanoflower, etc. have been employed with the aim of overcoming the aforementioned limitations [33]. Significant effort is being placed to employ these exotic materials into the POC applications [34]. This review emphasizes on the application of 2D materials in development of electrochemical POC devices for cancer diagnostics. Different types of 2D materials and their biomedical applications will be briefly introduced. Subsequently, their functions on the fabrication of electrochemical biosensors will be discussed. In addition, recently developed electrochemical POC devices for cancer diagnostics based on 2D materials and their future perspectives will be reviewed. 

## 4. 2D Materials and Their Biomedical Applications

2D materials are the class of substances that possess one nanoscale dimension (< 100 nm) with remarkable potential in various areas of electronics, optics, energy storage, chemical, and biomedical applications [35]. Extensive researches are being focused on developing various 2D structures for medical and biomedical applications, such as bio-imaging, photothermal therapy, drug delivery, cell differentiation, and diagnosis due to their anisotropic features, tunable functionalities, high surface to volume ratio, and unique physiochemical properties [36,37]. Graphene, as the first discovered 2D material with astonishing properties and successful implementation in biomedical technology, motivated the researchers to explore and find new 2D materials [38]. Up until now, different types of 2D materials have been introduced, such as carbon-based materials, hexagonal boron nitride (hBN) [39], black phosphorous (BP) [40], transition metal oxides (TMOs) and transition metal dichalcogenides (TMDs) [41], topological insulator bismuth selenide (Bi_2_Se_3_) [42], and more recently MXenes [43]. 

### 4.1. Carbon-Based 2D Materials 

As the most known carbon-based 2D material, graphene has long been used in diverse fields of diagnostics as being engaged in the development of implantable biosensors [44], optical and electrochemical sensors as well as cancer therapy, stem cell differentiation, and gene/drug delivery [45]. The high degree of biocompatibility of graphene has made this material a very successful candidate for healthcare applications. In addition, it has been shown that the integration of graphene with various nanomaterials can considerably improve their physiochemical properties. However, a lack of band gap in graphene has restricted its broad applications, particularly in semiconducting and electronics industry, where there is a need for device switching (e.g., logic gates and transistors) [46]. 

Various carbon-based 2D materials have been introduced, such as graphene oxide (GO), reduced graphene oxide (rGO), graphyne, graphane, fluorographene, and graphdiyne. Amongst them, GO and rGO have attracted much interest for biomedical applications due to their excellent hydrophobicity and surface functionalization abilities [47]. rGO can be obtained by a reduction of GO while using a straight-forward protocol to lower the high negative charge of GO, giving it more useful applications. Possessing OH and COOH functional groups on their surfaces, GO and rGO can be easily conjugated with different molecules/biomolecules for a variety of diagnostic and therapeutic applications [48].

### 4.2. Graphene-Like 2D Materials

hBN is relatively a new class of graphene-like 2D material with wide band gap. Its structure includes boron and nitrogen atoms covalently bound in a hexagonal lattice configuration on a same plain to form a layered structure. These layers are stacked on each other via weak van der Waals forces. Its tunable bandgap, high thermochemical stability, together with its high surface area, have made it a potential platform for drug loading and biosensor industry [39]. Another thermodynamically stable 2D material is BP (also known as phosphorene) with a orthorhombic structure that is very analogous to graphene, as it is black and flaky [49]. Similar to graphene, BP can be obtained while using the exfoliation (scotch-tape delamination) method. The highly anisotropic behavior of this puckered honeycomb structural material, alongside its sound biocompatibility, direct bandgap, light-induced biodegradability, as well as high thermal and charge transport features, have favored its biomedical applications in drug loading and diagnostics. However, the main concern that is faced by contemplating BP for biomedical applications is its gradual oxidization and formation of phosphoric acid when exposed by the oxygen in water [40]. 

Likewise, TMOs, such as Ag_2_O, NiO, CuO, and MoN_2_, are 2D materials of which the transition metals and oxygen atoms are strongly held together to represent interesting properties (optical, electrical, chemical, thermal, and physical) for application in magnetic resonance imaging (MRI), target delivery, etc. [50]. Despite the fascinating features of hBN, BP, and TMOs, they have not been widely explored in biotechnology unless in the form of nanosheets and composition with other materials, which will be reviewed later in this article.

### 4.3. Transition Metal Dichalcogenides (TMDs)

Transition metal dichalcogenides (TMDs) are composed of a layer of transition metals (e.g., tungsten, molybdenum, and niobium) sandwiched between two layers of chalcogen atoms (e.g., selenium or sulfur) that are covalently bound together [51]. Similar to graphene, the three-layered sheets are held on each other by weak van der Waals bonds. Although, single sheets of TMDs are not as thin as graphene nor mechanically stronger, they have displayed different electrical behavior, owing to their indirect bandgap. For instance, molybdenum disulfide (MoS_2_) and tungsten disulfide (WS_2_) are semiconductors; tungsten ditelluride (WTe_2_) and titanium diselenide (TiSe_2_) are semimetals; and, niobium disulfide (NbS_2_) and vanadium diselenide (VSe_2_) are conductor [52]. Interestingly, it has been reported that the bandgap of TMDs can be tuned as their thickness reduction is followed by the bandgap extension. This properties has interested various research fields to utilize TMDs in electrical, optical, and recently biomedical applications [53]. However, the widely-used TMD in diagnostic devices is MoS_2_, which has recently represented great potential in nanomedicine and biochemistry due to its high biocompatibility, ease of synthesis, and exceptional physiochemical properties, specifically at the nanoscale (single sheet).

### 4.4. Topological Insulator Bi_2_Se_3_

In 2011, the concept of topological insulator (TI) was introduced after observing exotic properties of Bi_2_Se_3_ to attract intensive interests from various interdisciplinary research fields [54]. TIs are electronic materials maintaining a bulk bandgap similar to ordinary insulators but protected conducting states on their surfaces. Although, some insulators have represented electrical current on their surfaces, but these currents are highly unstable and they tend to disappear under a small deformation of materials. TIs appeared to be a new class of quantum matters with conducting surface states and semiconducting bulk due to their strong spin-orbit interaction. These materials are so-called topologically protected, as such they are immune to small time-reversal perturbations, such as crystal disorders and backscattering [55,56]. In other words, the small corruption or contamination of TIs might not have a notable impact on their electrical properties. This might result in very steady physiochemical properties to make these materials very reliable for electronic and biomedical industry where the reproducibility is a must.

Topological insulator Bi_2_Se_3_ has exhibited very promising features, like Dirac plasmons, photothermal-conversion ability, thermoelectric behavior, exceptional electrochemical behavior, and unique electrical conductivity due to the presence of the single-surface Dirac cone and the six-valley degeneracy [57]. Different Bi_2_Se_3_ nanoparticles and nanosheets have been synthesized and applied in biotechnology to demonstrate great capability for clinical applications, such as cancer radiation therapy and imaging, cell differentiation, and biomarker detection [42,58]. TIs are critical rivals for graphene due to the (**i**) retention of their electron-state coherence up until room temperature (RT) together with the fact that, (ii) TI surface states insure a 2D Dirac system through a bulk material that is only observed in graphene at the atomic monolayer, far below the RT. However, low water dispersibility, difficult functionalization and relatively low biocompatibility of Bi_2_Se_3_ has restricted its biomedical applications. Although, Mohammadniaei et al. reported that the encapsulation of Bi_2_Se_3_ with gold can remarkably overcome those drawbacks [59,60], yet to explore more straight-forward methods.

### 4.5. MXene

Mxene is a newly discovered 2D material with very unique morphology, maintaining an accordion-like structure consisting of stacked 2D layers to provide a considerably high surface to volume ratio [61]. These layered structures are composed of transition metal carbides, carbonitrides, or nitrides with the general formula of M_n+1_X_n_T_x_, where M represents a transition metal atom, X refers to C and/or N atoms, T_x_ denotes the surface groups (e.g., O, OH, F, Cl), and n = 1, 2, or 3. Out of the different variety of introduced MXenes (~ 70 types), the Ti_3_C_2_T_x_ member has been studied more and applied in many fields of electrochemical energy storage, suppercapacitors, electromagnetic interference shielding, cancer therapy, and diagnostics [62,63]. Mxene Ti_3_C_2_T_x_ has displayed several advantages to be employed in biosensing field, such as (**i**) considerably high surface area for sufficient ligand loading and efficient electrocatalytic reaction; (ii) sound biocompatibility; (iii) good electrical conductivity for application in electrochemical biosensors; (iv) high abundance of surface functional groups; and, (**v**) the ability to passivate and resist against biofouling and non-specific bindings [64]. However, the stability of Mxene Ti_3_C_2_T_x_ as the solid electrode in aqueous solutions under different redox potentials has not been fully understood yet [65].

## 5. Graphene-Based Electrochemical Biosensors/Sensors for Cancer Diagnostics

Graphene, GO and rGO have been widely exploited in the development of electrochemical sensing devices due to their excellent catalytic and electrochemical features. In this section, different types of graphene-based biosensors and sensors for electrochemical detection of cancer biomarkers will be reviewed and their ability to realize POC screening will be discussed. Moreover, the role of graphene, GO, or rGO on the performance of each platform will be highlighted and compared with the other related platforms.

### 5.1. Graphene-Based Nucleic Acid Detection 

Cancer-related nucleic acids or oncogenic nucleic acids (circulating nucleic acids and microRNA) have been recognized as very promising biomarkers for early cancer diagnosis [66,67]. Circulating nucleic acids (ctNAs), which are released from lysed circulating tumor cells (metastatic tumor cells) into the patient blood stream, can be detected as the sign of cancer, even if the circulating tumor cells are not found. On the other hand, microRNAs (miRNAs) have gained much interest as the prospective candidates for cancer diagnosis due to their existence in all of the body fluids as well as their altered expression levels in healthy and cancer cells [68]. They are short non-coding RNA strands (~21 nucleotides) with very homologous sequences and low abundance to make their accurate detections challenging. Graphene-based electrochemical biosensors have offered very sensitive platforms for rapid, cost-effective, and selective quantification of different ctDNAs and miRNAs. For instance, in 2014, Rasheed et al. introduced a graphene-based electrochemical genosensor for the detection of breast cancer-related gene (BRCA1). The sensing mechanism was on the basis of a sandwich assay, in which two probes of capture and reporter were used to hybridize with the target gene and form a sandwich architect on a GO-modified glassy carbon electrode (GO/GCE). The reporter probe was labeled with gold nanoparticle to act as the redox dye for electrochemical detection of target gene. The proposed sensor offered a good sensitivity of 1 fM with linear detection range from 1 fM to 1 nM of the logarithmic target gene concentration due to the synergic effect of graphene and gold nanoparticle in the electrochemical signal enhancement [69]. 

**Figure 3:**
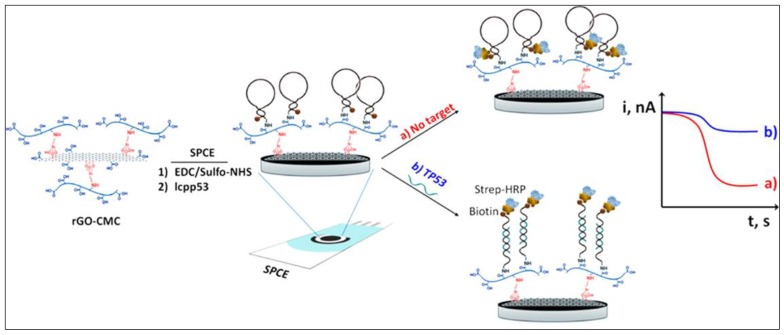
Schematic diagram for p53 tumor suppressor (TP53) gene detection based on reduced graphene oxide-carboxymethyl cellulose (rGO–CMC) nanohybrid scaffold. Reproduced with permission from [70].

Towards POC cancer screening, Pingarrón’s group proposed an electrochemical genosensor for p53 tumor suppressor (TP53) quantification in total patient serum [70]. As illustrated in Figure 3, disposable screen-printed carbon electrode (SPCE) was modified with rGO-carboxymethyl cellulose (rGO-CMC) to provide a highly conductive electrode and a suitable scaffold for being further functionalized by the hairpin capture probes that were priorly modified with amine group and biotin at their both ends. Upon the target hybridization, the hairpin probe was opened and the biotin was accessible to conjugate with the streptavidin peroxidase (Strep-HRP). 3,3′,5,5′-tetramethylbenzidine (TMB) and H_2_O_2_ were used as the redox mediator and enzyme substrate, respectively, for the amperometric detection of TP53 from breast cancer cell lines. This approach demonstrated a limit of detection (LOD) of 2.9 nM as well as the capability for the discrimination of single nucleotide polymorphism (SNP). 

Zhao et al. reported an ultra-sensitive electrochemical detection of CCND2 gene, which is overexpressed in various cancer types [71]. In this study, rGO was modified with gold nanoparticles-Fe_3_O_4_ nanocomposite and p-sulfonated calix [8] arene (Au@SCX8-RGO) to have supermolecular recognition ability and high catalytic activity. The electroactive toluidine blue (TB) molecule was loaded inside the SCX8 and the structure was functionalized with a label probe (LP) to form Au@SCX8-RGO-TB-LP bioconjugate. Upon the target invasion, the hybrid structure was conjugated to a capture probe modified with Au-Fe_3_O_4_ nanocomposite to form a sandwich assay. While using SPCE electrodes, the developed biosensor could detect CCND2 gene at the sub-aM concentration and single-cell level.

Graphene-based electrochemical biosensors have been also used to develop reliable, accurate and non-invasive electrochemical miRNA biosensors for cancer diagnosis. The first graphene-based electrochemical biosensor was introduced by Ai’s group, to which the GCE was modified with graphene nanosheets and further deposited by dendritic gold nanostructures (DenAu) to provide a highly conductive and electroactive electrode surface [72]. The electrode was then functionalized by locked nucleic acid (LNA)-integrated hairpin molecular beacon (MB) to form the capture probe. After the target miRNA hybridization, the MB (partially complementary to the target) was opened to form a sandwich architect with the bio-barcode gold nanoparticle Strep-HRP conjugate, followed by electrochemical detection via the chemical oxidation of hydroquinone by H_2_O_2_. A relatively low LOD of 60 fM was achieved by the proposed biosensor with the ability to analyze human hepatocarcinoma BEL-7402 cells and normal human hepatic L02 cells. 

**Figure 4:**
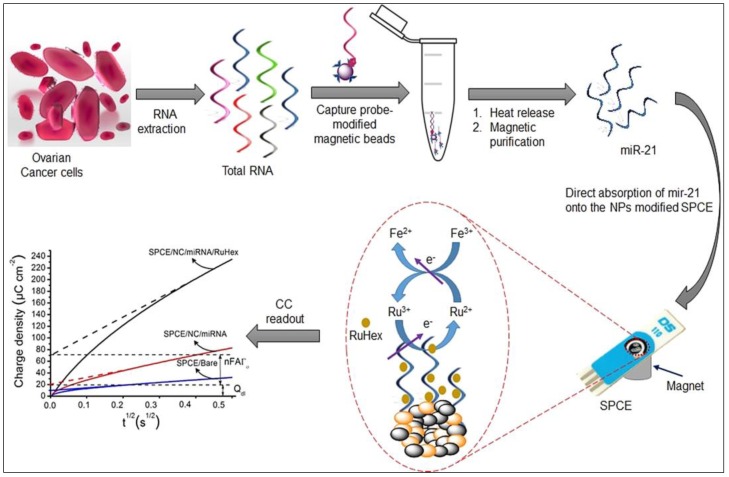
MicroRNAs (MiRNA) quantification assay on GO/IO- modified screen-printed carbon electrode (SPCE), based on intercalation of [Ru(NH3)_6_]^3+^ within the target miRNA and chronocoulometric detection while using [Ru(NH3)_6_]^3+^-Fe(CN)_6_]^3−^ couple. Reproduced with permission from [77].

To this end, many electrode modifications have been reported with the aim of developing sensitive graphene-based electrochemical biosensors for miRNA detection, such as rGO/carbon nanotube@screen printed gold electrodes [73], GO/gold nanorod [74], magnesium oxide nanoflower and GO/gold nanoparticles hybrids [75], sandwiched AgNPs in PANI and N-doped graphene [76], etc. However, to realize the POC cancer screening, Shiddiky’s group recently represented a very sensitive genosensor for detection of miRNA extracted from ovarian cancer [77]. As illustrated in Figure 4, a biotinylated capture DNA and streptavidin-dynabeads were used to purify the target miRNA-21 from the total RNA extracted from serum. The target miRNAs were purified, heat-released, and physically adsorbed on the graphene surface using superparamagnetic graphene-loaded iron oxide nanoparticles modified on SPCE (SPCE/GO-IO). It was followed by chronocoulometric quantification while using [Ru(NH_3_)_6_]^3+^ redox reporter and further amplification via ferricyanide redox system. The assay represented high sensitivity with bery good sensitivity (LOD = 1 fM) and potential application in clinical samples. Table 1 summarizes graphene-based electrochemical genosensors developed and employed for the quantification of cancer-related ctNAs and miRNAs.

### 5.2. Graphene-Based Protein Detection 

In fact, graphene-based electrochemical biosensors have been more practically applied in the development of protein biomarker sensors for POC cancer screening when compared with nucleic acids detection. In 2010, Du et al. fabricated the first graphene-based electrochemical immunosensor for the detection of α-fetoprotein (AFP) with LOD of 0.02 ng mL^−1^ and a linear range of 0.05 to 6 ng·mL^−1^ [83]. As the sensor platform, GCE-modified graphene sheet was modified by primary antibodies (Ab_1_) as the capture probe. The detection probe consisted of horseradish peroxidase (HRP)-secondary antibodies that were conjugated with carbon nanospheres (Ab_2_-HRP-CNS) to form a sandwich architect on the electrode upon the target detection. In the presence of H_2_O_2_, the electrocatalytic activity of HRP was recorded as the detection signal, while using the differential pulse voltammetry (DPV) technique. Graphene sheets displayed a seven-fold increase in the detection signal to pave the way in the development of various forthcoming graphene-based electrochemical immunosensors.

**Figure 5:**
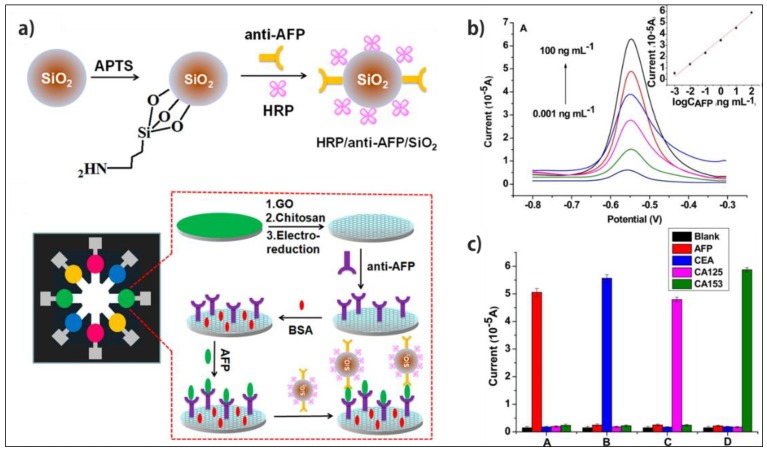
(**a**) Schematic illustration of fabrication of microfluidic paper-based analytical device (µPAD) for multiplex quantification of four cancer biomarkers; (**b**) Performance and the corresponding linear calibration curve of one platform toward different concentrations of AFP from 0.001 ng·mL^−1^ to 100 ng·mL^−1^; (**c**) electrocatalytic behavior of each platforms of (**A**) anti-AFP/rGO, (**B**) anti-CEA/rGO, (**C**) anti-CA125/rGO, and (**D**) CA153/rGO upon their matching target antigens of AFP, CEA, CA125, and CA153, respectively. Negligible cross reactivity was observed, showing the great specificity of the device. Reproduced with permission from [84].

Inspired by that, Hui’s group fabricated a remarkable µPAD for multiplex detection of four cancer biomarkers of AFP, carcinoembryonic antigen (CEA), cancer antigen 125 (CA125), and carbohydrate antigen 153 (CA153) [84]. They used GO that was electrochemically reduced onto the working electrode as the signal amplifier, biomarker-antibody attached onto the electrode as the capture probe and HRP/anti-biomarker /SiO_2_ nanobiohybrid as the detection probe (Figure 5). The DPV responses of the sandwich immunoreactions was obtained after target detection in the presence of *O*-phenylenediamine and H_2_O_2_. The developed device displayed very low LOD of 0.001, 0.005, 0.001, and 0.005 ng·mL^−1^ for AFP, CEA, CA125, and CA153, respectively. The fabricated device showed a great potential in POC cancer screening, owing to its low-cost, simplicity, and stand-alone ability to perform in real samples. 

With the benefit of outstanding electrochemical properties of graphene, many graphene-based electrochemical immunosensors have also been developed for specific and accurate detection of other cancer biomarkers, such as squamous cell carcinoma antigen (SCCA), folic acid protein (FP) [85], prostate special antigen (PSA) [86], human epidermal growth factor receptor 2 protein (HER2) [87], etc. However, in the direction with POC cancer screening, Kumar et al. fabricated cost-effective, flexible, and eco-friendly smart conducting paper device comprising a poly (3,4-ethylenedioxythiophene):poly(styrenesulfonate) (PEDOT:PSS) that was modified with rGO for the sensitive detection of CEA down to 25.8 µA·ng^−1^·mL·cm^−2^ [88]. 

**Figure 6:**
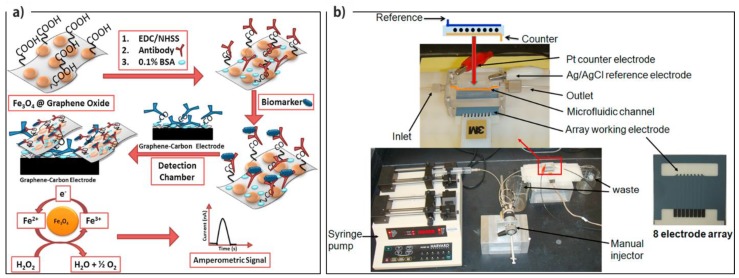
(**a**) Sandwich assay for quantification of prostate special antigen (PSA) and prostate specific membrane antigen (PSMA) based on antibody-modified Fe_3_O_4_@GO sheets on glassy carbon electrode (GCE) and further amperometric detection in the presence of H_2_O_2_; (**b**) Microfluidic device consists of an injector for delivering the biomarker captured Ab_2_@Fe_3_O_4_@GO to the detection compartment including an eight ERGO-coated electrode array, counter (Pt) and reference (Ag-AgCl) electrodes. Reproduced with permission from [89].

Later, Rusling’s group reported a very sensitive microfluidic-based mediator-free electrochemical device for the detection of PSA and prostate specific membrane antigen (PSMA) [89]. As illustrated in Figure 6, they used rGO-modified SPCE (ERGO) coated on an eight-electrode set as the sensing platform, which was later modified with primary antibodies. As the capture and detection probes, secondary antibody was conjugated onto Fe_3_O_4_ NP-grafted GO for the further amperometric detection of target protein in a sandwich assay. Fe_3_O_4_ NPs rolled as electrochemical signal generator in the presence of H_2_O_2_. The fabricated device represented very low LOD of 4.8 fg·mL^−1^ for PSMA and 15 fg.mL^−1^ for PSA in serum, which was about 1000 times lower than the previous methods, to be a sound candidate for the clinical diagnostics due to the synergic effect of two graphene derivatives (rGO and GO). Table 2 lists details regarding the other reports for electrochemical detection of protein biomarkers based on graphene and its derivatives. 

**Figure 7:**
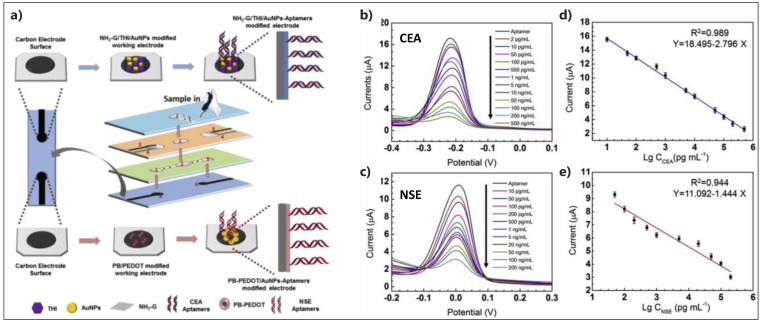
(**a**) Schematic design for fabrication and modification of µPAD for electrochemical detection of carcinoembryonic antigen (CEA) and neuron-specific enolase (NSE); (**b**,**c**) Sensor responses (DPV) to serial concentrations of CEA and NSE, respectively; and, (**d**,**e**) Corresponding calibration curves of peak currents as the function of logarithmic concentrations of CEA and NSE, respectively. Reproduced with permission from [90].

Recently, a newly-developed µPAD was reported for the simultaneous and label-free detection of two cancer biomarkers of CEA and neuron-specific enolase (NSE) in clinical samples [90]. The µPAD was fabricated based on screen printing and wax printing techniques, harboring two carbon working electrodes (Figure 7). The first working electrode was decorated by amino functional graphene-thionin-AuNP hybrid (NH_2_-G/THI/AuNP), and the second working electrode was modified by prussian blue-poly(3,4-ethylenedioxythiophene)-AuNP hybrid (PB/PEDOT/AuNP) to be further functionalized with two different aptamers, specific to the target proteins. Sensing mechanism was based on the recorded electrochemical signals of THI and PB in a signal-off manner, from which the formation of aptamer/antigen complex resulted in more surface coverage followed by hampering the electrochemical reaction at the solution/species interface. The hybrid nsnostructures displayed a double impact on increasing the (**i**) electron transfer rate and (ii) aptamer immobilization efficiency to give rise to the sensitive quantification of 2 pg·mL^−1^ CEA and 10 pg·mL^−1^ NSE, together with good linear ranges of 0.01–500 ng·mL^−1^ and 0.05–500 ng·mL^−1^, respectively. The proposed aptasensor demonstrated a very promising device for POC cancer screening.

### 5.3. Graphene-Based Small Molecule Detection 

Within the large number of small biomolecules, ROS and RNS have been more extensively studied as the potential candidates for cancer diagnosis. The abnormal levels of ROS and RNS are associated with several bodily disorders, such as Alzheimer, neurodegenerative diseases, and cancer due to their involvement in several physiological and cellular signaling pathways [7,102]. Among the ROS family, H_2_O_2_ has been widely studied as an important cancer biomarker. It has been reported that the stimulation of living cells with specific chemicals (e.g., N-for- myl-methionylleucyl-phenylalanine (fMLP), phobol 12-myristate-13-acetate (PMA), ascorbic acid (AA), adenosine 5’-diphosphate (ADP)) leads to considerable release of extracellular H_2_O_2_ from the cell membranes, which varies from healthy and cancer cells [103,104]. Nitric oxide (NO), on the other hand, as one of the RNS representatives with different important functions in biological signaling processes and pathological mechanisms, has demonstrated a critical biomolecule in tumor progression, neurotransmission, and carcinogenesis promotion [105]. 

Graphene has been employed to develop various enzymatic biosensors and non-enzymatic chemosensors for electrochemical quantification of H_2_O_2_ and NO in biological matrices owing to its high catalytic activity. Different enzymes have been incorporated with graphene to fabricate sensitive biosensors, such as horseradish peroxidase (HRP), ferredoxin (Fdx), myoglobin (Mb), hemoglobin (Hb), cytochrome c (cyt c), etc [11,106]. Typical reactions illustrating the electrocatalytic reduction of H_2_O_2_ and NO by Mb are shown, as follows:

For H_2_O_2_:

Mb(Fe^3+^) + e^−^ → Mb(Fe^2+^)

2Mb(Fe^2+^) + H_2_O_2_ → 2Mb(Fe^3+^) + 2H_2_O

For NO:

Mb(Fe^2+^) + NO → Mb(Fe^2+^)NO

2Mb(Fe^2+^)NO + 2H^+^ + 2e^−^ → 2Mb(Fe^2+^) + N_2_O + H_2_O

However, the non-enzymatic techniques have received intensive attentions for the construction of electrochemical devices due to the relatively low stability and high cost of the enzymes [107]. In 2010, Li’s group reported a smart multifunctional biointerface composed of a layered graphene-artificial peroxidase (AP)-extracellular matrix protein (Laminin) nanostructure for the electrochemical detection of H_2_O_2_ liberated from MCF-7 breast cancer cells upon PMA stimulation [108]. Graphene represented a significant bifunctional role to promote cell adhesion/growth and enhance the electrocatalytic activity of the chemosensor. The developed chemosensor showed a good sensitivity and great selectivity to motivate the exploration of graphene for development of various in situ and in vitro molecular electronic and cancer diagnostic devices.

Wu et al. also presented a sensitive non-enzymatic electrochemical sensor that is based on nitrogen-doped graphene on GC electrode for amperometric detection of H_2_O_2_ from neutrophils cells [103]. The nitrogen-doped graphene displayed much higher electrocatalytic activity than the graphene and/or GC to offer an LOD of ca. 0.05 µM. They also reported that the amount of released H_2_O_2_ from the cells was stimuli type-dependent, of which PMA showed a higher effect following by consecutive decrease in the order of fMLP, AA, and ADP. One year later, Li et al. synthesized a potassium (**K**)-modified graphene for amperometric detection of nitrite (NO_2_^−^), which is derived from NO and it is produced by NO synthase in wide range of cell types [109]. The K-modified graphene played the role of an electron transfer medium to promote the electrocatalytic activity of graphene, which leads to the sensitive detection of nitrite (LOD: 0.2 μM; linear range: 0.5 μM to 7.8 mM) and the ability of the chemosensor in monitoring extracellular NO_2_^−^ from liver cancer and leukemia cells. Lately, N and S dual-doped graphene (NSG) was reported to display pronounced electrocatalytic activity, sound durability, large electroactive surface area, and good biocompatibility to successfully profile two breast cancer cells (MDA-MB-231 and MCF-7) and Hela cells that are based on their flux of extracellular H_2_O_2_ [110]. 

Many researches have been conducted using graphene nanosheets and rGO modified with various catalytically-active nanostructures and/or enzymes to further improve the performance of electrochemical quantification of ROS/RNS in situ and *in vitro*. For example, graphene-decorated nanoparticles, such as Au [111], Pt [112], CuS [113], Fe_3_O_4_ [114], Ag [115], Cu [116], etc., have been successfully employed for monitoring H_2_O_2_ level in body fluids (human serum and urine) and released from different cancer cells (lung cancer A549, liver cancer HepG2, and glioblastoma cell U87), which provided very useful information regarding the progress of tumor development. Additionally, towards the fabrication of implantable devices, Duan’s group developed a flexible sensor using rGO paper that was decorated with Au@Pt core-shell nanoparticles to form a 2D closely-packed-assemblies for screening the NO secretion by human umbilical vein endothelial cells (HUVECs) after being stimulated by acetylcholine (Ach) [117]. Another graphene-based flexible paper device was introduced by the same group for real-time monitoring of extracellular H_2_O_2_ that was released by live cells macrophages [118]. In this approach, the rGO paper was decorated with Pt nanoparticles and carbon nanotubes (Pt/graphene–CNT paper) to exhibit very high sensitivity down to 10 nM, due to the synergetic contribution between the utilized nanomaterials to improve the electrical conductivity, mechanical strength, and surface area of the electrode. 

**Figure 8:**
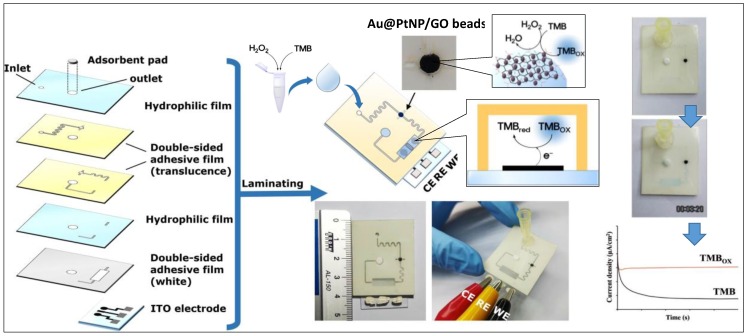
The fabrication procedure of microfluidic paper-based device and further electrochemical detection of H_2_O_2_ based on its reduction on Au@PtNP/GO nanozymes to oxidize 3,3′,5,5′-tetramethylbenzidine (TMB) in 1 min. The oxidized TMB was subsequently migrated to the working electrode for being reduced on ITO substrate and further detected using amperometric technique. Reproduced with permission from [119].

However, to realize the POC cancer screening while using graphene-based small molecule detection, very recently an electrochemical microfluidic device was fabricated by Ko et al. for monitoring the H_2_O_2_ level in artificial urine based on Au@Pt nanoparticle/GO microbeads [119]. As demonstrated in Figure 8, for the sensing performance, injection of TMB/H_2_O_2_ onto the device was followed by a rapid TMB_ox_ generation (1 min.) on the peroxidase-like Au@Pt nanoparticle/GO microbeads to be further reduced on the ITO electrodes and detected using chronoamperometric technique. The proposed work offered a sensitive (LOD = 1.62 µM; Linearity = 1 μM–3 mM), selective, portable, disposable, and inexpensive polymeric film-based device that provided inspiring results toward the understanding of electrochemical POC cancer screening based on the small molecules detection. Table 3 provides a comprehensive study on different reported sensors for H_2_O_2_ and NO detection.

## 6. MoS_2_-Based Electrochemical Biosensors/Sensors for Cancer Diagnostics

Distinctive physiochemical properties of MoS_2_, such as high conductivity, fast charge transfer, and large surface to volume ratio, have made it an excellent choice for electrochemical biosensing applications. Superior charge mobility and abundant active sites of MoS_2_ facilitates electron transportation between the conductive additives (graphene, carbon nanotube, Au, Pt, Ag, ect.) accommodated within the MoS_2_ layers to elevate its mechanical and electrochemical properties [126,127,128]. The integration of MoS_2_ with functional nanomaterials has resulted in the development of various electrochemical biosensors and sensors for sensitive detection of environmental pollutants, phenolic compounds, virus, bacteria, glucose, neurotransmitters, fatty acids, cancer biomarkers, etc [129]. In this section, recent advances in cancer biomarkers detection using MoS_2_-based electrochemical devices will be reviewed and those with the potential ability to realize POC screening will be highlighted. 

### 6.1. MoS_2_-Based Nucleic Acid Detection 

Wang et al. reported a label-free electrochemical biosensor that was composed of a layered MoS_2_−thionin composite for monitoring circulating DNA in human serum [130]. Upon DNA intercalation and electrostatic interaction with thionin, the redox current peak that was attributed to the thionin dropped, which indicated the prosperous potential of MoS_2_ for sensing and clinical diagnosis applications. Another approach was conducted for the electrochemical detection of ctDNA (related to E542K gene of gastric cancer) while using [Fe(CN)_6_]^3−^ as a redox label [131]. After the addition of ctDNA, ssDNA (probe), which was physically adsorbed on the MoS_2_/graphene nanocomposite/GCE, transformed to dsDNA to entrap more [Fe(CN)_6_]^3-^ ions, resulting in a higher redox current peak. The fusion effect of graphene and MoS_2_ provided an excellent platform for (**i**) anchoring the ssDNA (probe) via van der Waals bonding and (ii) ultrasensitive detection of ctDNA down to 10 fM. 

In another study by Zhang et al., ctDNA related to PIK3CA gene (breast cancer) was electrochemically monitored in peripheral blood while using MoS_2_ nanosheets that were functionalized with poly-xanthurenic acid (PXA) film. The probe ssDNA was non-covalently adsorbed onto the PXA via π–π interaction. After the target ctDNA hybridization, the resulted dsDNA was released from the PXA/MoS_2_ nanocomposite surface followed by a self-signal regeneration of the nanocomposite (signal-on). The fabricated sensor offered a pronounce sensitivity of 18 aM and linear range from 100 aM to 100 pM. A similar study was conducted by the same group for PIK3CA gene detection using MoS_2_ nanosheets functionalized with poly(indole-6-carboxylic acid) (PIn6COOH) film. The abundance of carboxyl groups on the PIn6COOH film provided proper covalent bonding with amine-modified ssDNA (probe). After the target binding, the dsDNA hampered the electrocatalytic activity of the PIn6COOH, leading to the self-signal drop of the nanocomposite (signal-off). MoS_2_ dramatically improved the electrochemical properties of PIn6COOH to give rise to an ultra-sensitive detection of 15 aM ctDNA and linear range from 100 aM to 10 pM. Both of the reported methods represented ultra-sensitive, simple, label-free, and single-step principle to be further studied for more practical approaches.

MoS_2_ has also shown a great feasibility for miRNA sensing applications (listed in Table 4). For instance, the MoS_2_ nanosheets were decorated with AuNPs (AuNP@MoS_2_) and either modified with capture DNA probes or detection DNA probes [132]. After the target (miRNA-21) hybridization, a sandwich structure was formed on the GCE for further analysis while using DPV and EIS methods. With good specificity towards single-mismatch mutations and the LOD of 0.78 fM as well as the detection linearity of 10 fM to 1nM, the proposed biosensor represented the prospective capability of MoS_2_ for the accurate detection of miRNAs. In a similar study, MoS_2_ nanosheet was functionalized with thionine and AuNPs (MoS_2_-Thi-AuNPs) for label-free electrochemical detection of miRNA-21 in human serum [133]. Later, Huang’s group constructed AuNP/hollow MoS_2_ microcubes for ultrasensitive quantification of miRNA-21 [134]. Sensing mechanism was based on the hybridization of target with biotinylated ssDNA immobilized on AuNP/MoS_2_ and further signal amplification while using duplex-specific nuclease (DSN) enzyme via electrochemical–chemical–chemical (EEC) redox cycling. Coupling the enzymatic signal amplification and MoS_2_ nanohybrid as the electrochemical signal booster resulted in a remarkable low LOD of 86 aM. Another report by same group was conducted for miRNA-21 detection (LOD = 16 aM) while using carbon sphere-MoS_2_ and catalyzed hairpin assembly, from which the enzymatic amplification in their previous work was replaced with an enzyme-free hybridization chain reaction method to reduce the production cost of the sensor [135]. 

**Figure 9:**
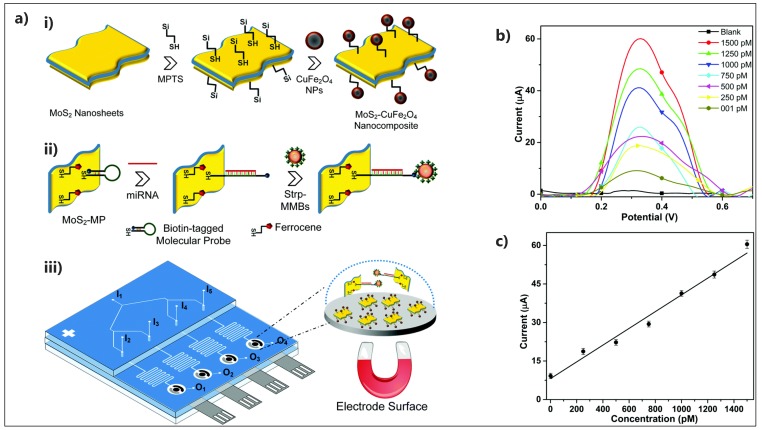
(**a**) Schematic illustration of (**i**) MoS_2_–CuFe_2_O_4_ nanocomposite synthesis, (ii) step-wise detection of target miRNA while using MoS_2_-MP nanocarriers and (iii) fabricated microfluidic platform consists of inlets (I_1_-I_5_), reaction and sensing zones and outlets (O1-O5); (**b**) Detected DPV signals for various concentrations of nominal miR-205 and (**c**) the corresponding linear curve for the obtained DPV data. Reproduced with permission from [141].

However, two device types have been fabricated thus far in regard to the MoS_2_-based electrochemical POC cancer screening. A paper-based device was developed for te simultaneous detection of miRNA-141 and miRNA-21 on a single electrode down to 100 aM [143]. The sensing performance was based on a sandwich assay consists of a hairpin DNA immobilized on MoS_2_/AuNPs/AgNW, target miRNA and detection probes (methylene blue (MB) or ferrocene (Fc)-labeled PtCuMOFs/DNAs). Although the device displayed the advantages of low cost and disposability, complicated detection mechanism as well as the tedious electrode preparation procedure restrict its clinical applications. Another approach was reported by Neethirajan’s group for MoS_2_-based electrochemical POC device coupled with a microfluidic device for the multiplex detection of four miRNAs [141]. As seen in Figure 9, two sets of MoS_2_ nanosheets were prepared. For the electrode surface modification, MoS_2_-CuFe_2_O_4_ nanocomposite was electro-polymerized on SPCE to serve as a highly electrocatalytic electrode due to the co-existence of MoS_2_ and Cu. The capture probe consisted of a biotinylated molecular probe (MP) and ferrocene co-immobilized on MoS_2_ nanosheets. After miRNA hybridization with the biotinylated MP, the closed loop was opened, which disentangled the biotin to be accessible and conjugated with streptavidin-coated magnetic microbeads (Strp-MMBs). Using an external magnetic field on the microfluidic device, the hybrid nanocomplexes were accumulated on the SPCEs followed by a detectable electrochemical signal arising from ferrocene. The proposed device represented an affordable and portable biosensing microfluidic platform with high sensitivity (LOD = 480 fM) and selectivity, thanks to the synergic function of MoS_2_ in signal enhancement and chemical loading elevation, to be a potential candidate for POC cancer screening. 

### 6.2. MoS_2_-Based Protein Detection 

The cooperative effects between nanomaterials and MoS_2_ has led to the development of several protein biomarker detection assays. Spacious layered structure of MoS_2_ can provide a great platform for the grafting and immobilization of different kinds of antibodies and biomolecules for the fabrication of many immunosensors. Huang’s group fabricated an electrochemical sandwich assay while using AuNPs/MoS_2_/carbon aerogel (CA) composite for the detection of platelet-derived growth factor BB (PDGF-BB), which is a cancer-related biomarker due to its involvement in genetic alterations and tumor progression/growth in many cancer types [144]. AuNP was co-functionalized with aptamer-specific PDGF-BB and Fc to be further coupled with the MoS_2_/CA-modified 2nd aptamer, after the target conjugation. The proposed assay showed a good sensitivity (LOD = 0.3 pM) and demonstrated sound stability and selectivity owing to its dual signal amplification system. MoS_2_ has been also employed for the quantification of cancer biomarker thrombin (TB) in human serum [145]. Palladium nanoparticles decorated graphene-molybdenum disulfide flower-like nanocomposites was constructed and coupled with glucose oxidase for th enzymatic detection of TB based on the H_2_O_2_ reduction on a hemin/G-quadruplex hybrid. MoS_2_ was used to enhance the graphene surface area and accelerate the charge transfer. A synergetic electrocatalysis was observed from the hybrid nanocomposite to give rise to the high sensitivity of the fabricated immunosensor (LOD = 0.062 pM).

Moreover, MoS_2_ nanocomposites have been widely applied for the electrochemical detection of CEA cancer biomarker. For example, an enzymatic sandwich assay was fabricated based on the catalytic activity of MoS_2_-Au nonocomposite using glucose oxidase-modified Ag nanosphere as the label. An LOD of 0.27 pg·mL^−1^ was achieved together with a linear detection range from 1 pg·mL^−1^ to 50 ng·mL^−1^ [146]. A simpler mechanism was reported by Lianhui Wang’s group based on AuNP-decorated thionine-MoS_2_ (AuNP-Thi-MoS_2_) for single-step, label-free, and accurate electrochemical detection of 0.52 pg·mL^−1^ CEA with a linear range from 1 pg·mL^−1^ to 10 ng·mL^−1^ [147]. The same group constructed a Prussian blue nanocube-decorated MoS_2_ nanocomposite for the dual detection of CEA (label-free) and H_2_O_2_ (non-enzymatic) with high sensitivity of 0.54 pg·mL^−1^ and 4.1 nM, respectively [148]. The MoS_2_ nanosheet was not only utilized for its high catalytic activity, but also for its great capability to stabilize Prussian blue nanocubes. To increase the sensitivity of their previous biosensors, they recently reported an ultrasensitive MoS_2_-based immunosensor for the enzymatic detection of CEA down to 1.2 fg·mL^−1^ with a wide range of 10 fg·mL^−1^–1 ng·mL^−1^ [149]. Target CEA was sandwiched between two layers of MoS_2_-AuNPs and HRP-mediated MoS_2_-AuNPs for further detection based on the o-phenylenediamine (o-PD) catalysis in the presence of H_2_O_2_. The high performance of the immunosensor was attributed to a triple signal amplification mechanism, of which (**i**) MoS_2_-AuNPs demonstrated efficient enzyme mimics and unique conductivity to facilitate o-PD/H_2_O_2_ reaction as well as (ii) a capacious structure for loading large numbers of HRP-anti-CEA and anti-CEA, also (iii) the introduction of HRP could block the nonspecific bindings. However, a similar performance was observed from a label-free immunosensor that was developed by Wei’s group with simpler design based on flower-like Ag/MoS_2_/rGO nanocomposites. Due to the contributory effects of the three electroactive structures, the fabricated immunosensor showed a high efficiency (LOD = 1.6 fg·mL^−1^; linearity = 10 fg·mL^−1^–100 ng·mL^−1^) and acceptable selectivity and reproducibility with the ability to perform in serum samples. 

**Figure 10:**
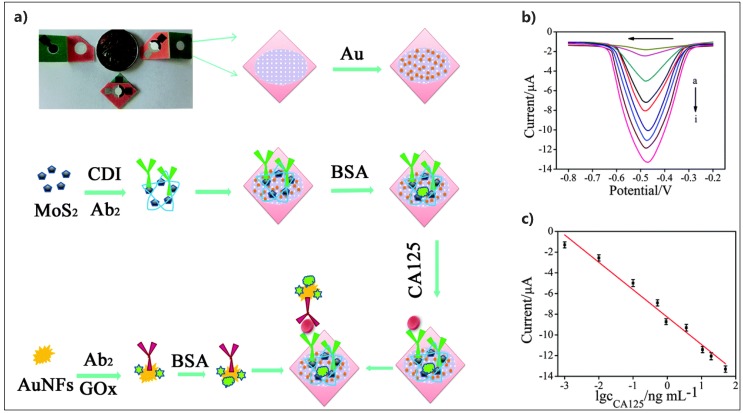
(**a**) Optical picture of the three-dimensional (3D) origami electrochemical immunodevice and the schematic modification and target detection procedure; (**b**) differential pulse voltammetry (DPV) response of the biosensor toward various concentration of CA125 in PBS (pH 7.6) containing 1% glucose; (**c**) Corresponding calibration curve of the detected peak current as the function of logarithmic concentration of CA125. Reproduced with permission from [150].

However, in the direction of POC cancer screening, Yan’s group fabricated a cost-effective microfluidic three-dimensional (3D) origami electrochemical device for sensitive detection of cancer antigen CA125 [150]. Au nanoparticle-modified paper electrode was constructed and functionalized with MoS_2_ nanosheets and capture antibodies to form a sandwich assay with Au nanoflowers (AuNFs) that were co-functionalized with glucose oxidase (GOx) and secondary antibody, after the target binding (Figure 10). MoS_2_ nanohybrid enhanced the catalytic activity of GOx toward H_2_O_2_ to fulfill the appropriate sensitivity of 0.36 pg·mL^−1^. The fabricated device represented a suitable platform for the application of electrochemical POC cancer screening that is based on MoS_2_ composites. Table 5 provides details for the other reported literatures for MoS_2_-based electrochemical detection of protein cancer biomarkers. 

### 6.3. MoS_2_-Based Small Molecule Detection

MoS_2_ has been also employed for the non-enzymatic/enzymatic detection of H_2_O_2_ and NO (NO_2_^−^) due to its high electron mobility to lower the response time and widen the saturation time of the biosensor. A peroxidase-like activity toward H_2_O_2_ reduction has been observed from the MoS_2_ nanoflakes with and without modification with inorganic nanostructures or biomolecules [32]. However, the modification of MoS_2_ has remarkably enhanced its electrocatalytic activity. 

#### 6.3.1. Non-Enzymatic Detection

Several MoS_2_-related nanocomposites have been synthesized for the non-enzymatic detection of H_2_O_2_ in PBS and spiked serum, such as flower-like MoS_2_/rGO with the LOD of 25 nM [156], 3D Cu nano-flowers/layered MoS_2_, as well as Au-Pd/MoS_2_ composites for the dual detection of H_2_O_2_ and glucose [157,158], hierarchical oxygen-implanted MoS_2_ nanoparticle decorated graphene for high sensitivity of 269.7 μA·mM^−1^·cm^−2^ [159], Prussian blue-MoS_2_-rGO for rapid detection (< 10 s) [160], 3D rGO-MoS_2_-QDs with wide detection range of 10 µM to 5.57 mM [161], etc., (refer to Table 6 for more details and the other reports). 

**Figure 11:**
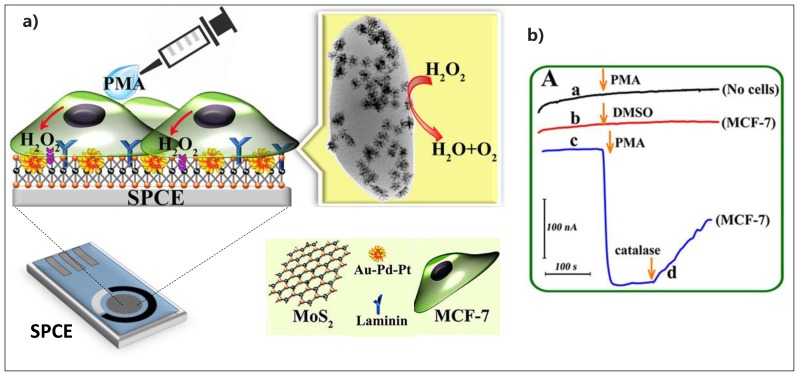
(**a**) Schematic diagram for in-situ screening of the released H_2_O_2_ from MCF-7 breast cancer cell lines based on SPCE-modified Au−Pd−Pt/MoS_2_ nanoflower-dispersed nanosheets; (**b**) Amperometric response of the fabricated chemosensor to addition of PMA in the absence and presence of cultured cells as well as DMSO as the control. Reproduced with permission from [165].

A bimetallic nanocube platinum-tungsten (PtW) was synthesized and self-assembled onto the MoS_2_ nanosheets for the quantification of H_2_O_2_ secreted from mouse breast cancer cells for the detection of extracellular H_2_O_2_ from cancer cells (cell line: 4T1) [162]. With an ultra-sensitivity (LOD = 5 nM) and high selectivity towards different interfaces including ascorbic acid (AA) and uric acid (UA), dopamine (DA), Hb, paracetamol (4-acetamidophenol, APAP), NO_3_^−^, and K^+^, the proposed platform represented the great capability of MoS_2_ for H_2_O_2_ monitoring in living cells. Dai et al. reported an electrochemical for monitoring H_2_O_2_ released from lung cancer cells (cell line: A549), based on MoS_2_/nitrogen-doped carbon nanowires (MoS_2_/C_N_ NWs) [163]. The electrochemical catalytic performance of the chemosensor was improved because of the abundant active sites of MoS_2_ nanosheets as well as the high stability of the C_N_ NWs framework. In another study, MoS_2_ nanosheets were decorated by Au nanorods and then modified onto the microneedles for real-time monitoring of H_2_O_2_ released from Hela cells [164]. By taking the advantage of acupuncture needles, the authors developed a simple, affordable, and novel platform for electrochemical detection of H_2_O_2_ to be further studied for screening the bioactive molecules *in vivo*. Recently Dou et al. reported in situ monitoring of endogenous H_2_O_2_ secreted from MCF-7 human breast cancer cells, based on direct and non-invasion electrochemical measurement on the disposable SPCE-modified Au−Pd−Pt/MoS_2_ nanoflower-dispersed nanosheets [165]. As seen in Figure 11, the cells were adhered and then grown on the electrode with the help of the pre-immobilized laminin glycoproteins and treated with PMA for further electrochemical analysis. The trimetallic hybrid nanoflowers and the MoS_2_ nanosheets provided a synergetic effect to amplify the electrocatalytic activity of chemosensor and achieve an ultra-low LOD of 0.3 nM.

The non-enzymatic electrochemical detection of NO and NO_2_^−^ has been also reported while using MoS_2_-based approaches, however the analytical performance of the developed chemosensors on cancer cells have not been thoroughly studied. For instance, GCE was decorated with flower-like Fe_2_O_3_@MoS_2_ nanocomposite for the amperometric detection of NO_2_^−^ [166]. A wide detection range from 2 to 6730 µM, together with a high sensitivity of 1 µM, was observed from the developed chemosensor. Wang et al. reported a straw cellulose/MoS_2_ nanocomposite drop-casted on GCE for the accurate detection of NO_2_^−^ [167]. The fabricated chemosensor was cost-effective, sensitive (LOD = 2 µM), and very selective to determine NO_2_^−^ in the presence of different interfaces (Cl^−^, Cu^2+^, SO_4_^2−^, NO^3−^, Al^3+^, and Fe^3+^ in 100-fold concentrations). In another study, AuNPs were decorated onto the MoS_2_ nanosheets for simultaneous detections of DA, AA, UA, and NO_2_^−^ [168]. A similar approach was recently used for the decoration of 3D flower-like MoS_2_ with AuNP to monitor NO_2_^−^ in real samples [169]. The proposed platform showed a high sensitivity (117.0 µA·mM^−1^·cm^−2^) and low LOD (1.67 µM) together with good stability, selectivity, and reproducibility.

#### 6.3.2. Enzymatic Detection 

**Figure 12:**
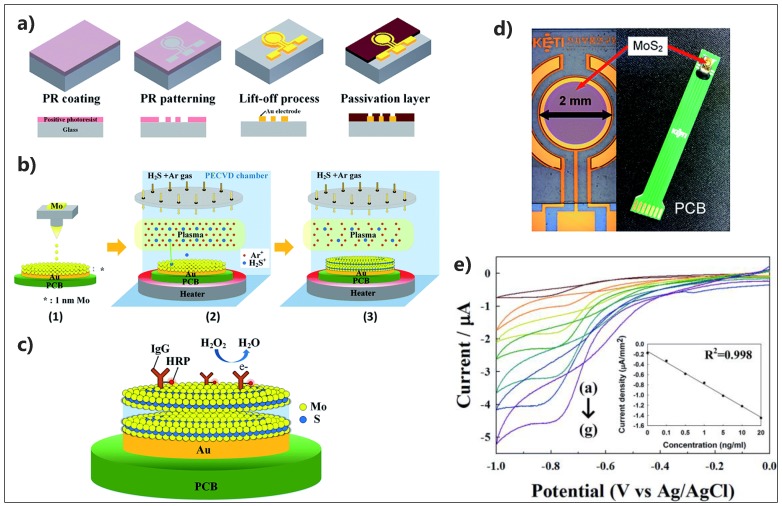
Schematic diagram of (**a**) Screen-printed electrode fabrication process; (**b**) In situ growth of MoS_2_: (1) deposition of 1 nm Mo on gold using e-beam evaporator, (2) reaction of Mo with H_2_S + Ar plasma and (2) formation of MoS2 on gold electrode in a plasma enhanced chemical vapor deposition (PECVD) chamber; (**c**) Presentation of MoS_2_-HRP-IgG biosensor for H_2_O_2_ detection; (**d**) Optical image of the fabricated device; (**e**) Device performance upon different concentrations of HRP-IgG (**a**) 0, (**b**) 0.1, (**c**) 0.5, (**d**) 1, (**e**) 5, (**f**) 10; and, (**g**) 20 ng·mL^−1^. Reproduced with permission from [170].

Kim et al., reported the first enzymatic MoS_2_-based biosensor for H_2_O_2_ detection with promising potential for POC screening applications [170]. The device, polymeric printed circuit board (PCB), was fabricated with Au electrodes electroplated on the surface. The MoS_2_ layers were nucleated on the working electrode using plasma enhanced chemical vapor deposition (PECVD) technique (Figure 12). Different concentrations of HRP-IgG (0–20 ng·mL^−1^) were later immobilized on the modified electrode to evaluate the H_2_O_2_ response of the device in the presence of hydroquinone as the mediator. The device performance was simple and it required very low sample volume to be a practical method for the further development of commercially available POC devices. 

Later, the AuNP-decorated MoS_2_ nanosheet (AuNPs@MoS_2_) was employed and immobilized by Hb for the further quantification of H_2_O_2_ and NO [171]. The electroactive prosthetic group of Hb (Fe^2+^) as well as the high performance of AuNPs@MoS_2_ nanocomposite to provide a biocompatible 3D platform for efficient immobilization and excellent bioelectrocatalytic activity of Hb, resulting in the sensitive detection of both H_2_O_2_ and NO with LOD of 4 and 5 µM, respectively. Similar approach was used for the amperometric detection of H_2_O_2_ in SP2/0 mouse myeloma cancer cells based on MoS_2_ nanosheet–Au nanorod hybrids on GCE to immobilize catalase enzyme [164]. A highly sensitive biosensor was achieved with LOD of 100 nM and a linear range of 0.5 to 200 µM. A notable selectivity toward glucose (GLU), AA, DA, UA, salicylic acid (SA), and glycine, L-cysteine was observed, which illustrated the acceptable performance of the fabricated biosensor. 

In another study, Layered MoS_2_–graphene composites was used to immobilize Mb and subsequently detect H_2_O_2_ [172]. A relatively lower LOD (1.25 µM) was observed which would be as a result of fusion effects of the two 2D materials. Likewise, Yoon et al. reported a very sensitive electrochemical biosensor for NO detection based on amine-modifiedMoS_2_/GO/Mb hybrid [173]. The amine-modified MoS_2_ nanoparticles were encapsulated by GO via amide bonding. The hybrid structure was covalently immobilized on the Au electrode while using cysteamine chemical linkers and further modified by Mb. The developed biosensor represented an ultra-sensitivity of 3.6 nM and very good reproducibility. 

## 7. Bi_2_Se_3_-Based Electrochemical Biosensors

In 2012, Fan et al. developed the first enzymatic Bi_2_Se_3_-based electrochemical biosensor for H_2_O_2_ quantification [186]. Flower-like Bi_2_Se_3_ nanostructures were synthesized for direct electrochemistry of Hb towards H_2_O_2_ reduction while using a facile hydrothermal technique. The Bi_2_Se_3_ nanostructures were first assembled on the GCE via Nafion, then the Hb proteins were immobilized on the substrate. The Bi_2_Se_3_ nanostructures significantly promoted the electrocatalytic activity of Hb, due to their special morphology to properly entrap Hb and their excellent charge transfer properties. The developed biosensor represented a high sensitivity down to 0.63 uM and a sound stability, also provided a promising matrix for protein immobilization. Bi_2_Se_3_ was also used for the development of IgG electrochemical immunosensor [187]. In this approach, the Bi_2_Se_3_ nanosheets were modified onto carbon paste electrode (CPE) via ionic liquid ([BMIm]BF_4_ IL) as the sensing interface. This was followed by immobilization of anti-human immunoglobulin G (anti-IgG) while using glutaraldehyde (GA) crosslinking. The fabricated biosensor represented high sensitivity and specificity with LOD of 0.8 ng·mL^−1^ and linear detection range of 2–300 and 300–2200 ng·mL^−1^, demonstrating the potential application of Bi_2_Se_3_ in biomarker quantification. 

**Figure 13:**
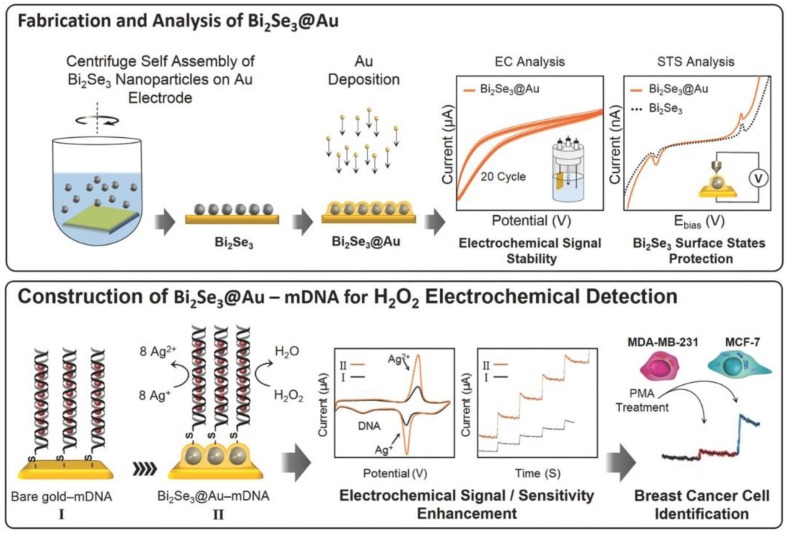
Schematic for stepwise fabrication and analysis of Bi_2_Se_3_@Au electrode as well as construction and performance of Bi_2_Se_3_@Au-metallic DNA biosensor toward profiling two breast cancer cells based on their H_2_O_2_ abundance. Reproduced with permission from [60].

Recently, Mohammadniaei et al. reported an ultra-fast and highly sensitive Bi_2_Se_3_-based electrochemical biosensor to profile two different breast cancer cell lines of MCF-7 and MDA-MB-231 based on the abundance difference of their extracellular H_2_O_2_ [60]. As displayed in Figure 13, the synthesized Bi_2_Se_3_ were sandwiched between two layers of gold, which provided a very robust platform for further modification of Ag^+^-modified DNAs via the straight-forward thiol-Au bonding. Using the scanning tunneling spectroscopy technique, the authors proved that the encapsulation of Bi_2_Se_3_ with thin layer of Au preserved the TI surface states, improved its electrochemical-signal stability, and amplified the electrochemical signal of Ag^+^ by ca. 10-fold of magnitude. The developed biosensor showed an ultra-fast response (1.6 s) and significantly low LOD of 10 nM with considerable capability to work in real samples and discriminate between two cancer cell lines. The authors also revealed that the Bi_2_Se_3_ served not only as the signal booster, but also as the noise reductant to considerably ameliorate sensor performance. 

In spite of the very promising features of the Bi_2_Se_3_, however, a handful of studies have been conducted on the Bi_2_Se_3_-based electrochemical biosensors. Although, Bi_2_Se_3_ has a great potential to open a new horizon toward upgrading the existed electrochemical biosensors due to its observed exotic properties, such as (**i**) the ability to damp the backscattering/fluctuation of the spin-polarized charges existed on its surface states, resulting in lowering the electronic noises and increasing the signal-to-noise ratio; (ii) Having a topologically-protected surface to improve the reproducibility challenges that are faced by nanoparticle applications; and, (iii) possessing a 2D Dirac system to represent a high conductivity and charge mobility at room temperature. 

## 8. MXene-Based Electrochemical Biosensors

MXene nanosheets have been applied for electrochemical detection of cancer biomarkers, as the biocompatible transition metal structures. Very special morphology of MXenes enables them to efficiently entrap and immobilize various enzymes, metalloproteinase and nucleic acid structures for direct electron transfer following by supreme electrochemical activities. Liu et al. immobilized Hb on synthesized MXene-Ti_3_C_2_ nanosheets for nitrate detection with an LOD of 0.12 µM and a broad linear range from 0.5 to 11800 µM [188]. A similar structure was used by Wang et al. to quantify H_2_O_2_ within the linear range of 0.1–260 µM and very low LOD of 20 nM. [189].

The high performance of MXene-Ti_3_C_2_ as an exceptional immobilization matrix and an excellent charge transport accelerator inspired further studies. TiO_2_ nanoparticles were incorporated into the MXene-Ti_3_C_2_ flakes to improve the performance of MXenes towards (**i**) increasing its surface area and efficient engulfment of redox Hb proteins, (ii) biocompatibility enhancement, and (iii) long-term stability of biosensor for two months [190]. The fabricated H_2_O_2_ biosensor represented a high sensitivity of 447.3 μA·mM^−1^·cm^−2^ and LOD of 14 nM. In another study, the MXene-Ti_3_C_2_/GO nanocomposites were synthesized and used to immobilize Hb for further application in inkjet-printed hydrogen peroxide (H_2_O_2_) biosensing [191]. The printed biosensor showed a dynamic range of 2 μM-1 mM and an LOD of 1.95 μM, with high sensitivity and selectivity.

MXene has also been employed for the electrochemical detection of CEA cancer biomarker [192]. MXene-Ti_3_C_2_ was drop-casted onto GCE and subsequently functionalized using aminosilane APTES for covalent bonding with anti-CEA. Using redox probe hexaammineruthenium ([Ru(NH_3_)_6_]^3+^), the target CEA was quantified within the linear range of 0.0001 to 2000 ng·mL^−1^, very low LOD of 18 fg·mL^−1^, and sensitivity of 37.9 μA·ng^−1^·mL·cm^−2^. The performance of the proposed biosensor was quite comparable with other 2D material-based biosensors, however this approach was simpler and more cost-effective. Liu et al. recently developed a microRNA biosensor for electrochemical quantification of miRNA-128 (lung cancer biomarker) using AuNP/MoS_2_/Ti_3_C_2_ nanohybrids [142]. The fusion effect of the two 2D materials provided a great electroactive platform for immobilization of thiolated ssRNAs (probe) onto the AuNPs. After the target hybridization with probe, the dsRNAs were released from the surface due to the suggested swelling-induced breakage of Au–thiol bonds between RNA and the electrode surface. This was followed by reaching more redox probe [Fe(CN)_6_]^3−/4−^ to the surface and increment in the detected electrochemical signal. A very low LOD of 0.43 fM and wide detection range from 1 fM to 0.1 nM was claimed by the authors. Although, due to the new emergence of MXenes in electrochemical biosensing mechanisms, more comprehensive studies are required to understand the practical applications of these interesting materials.

## 9. Electrochemical Biosensors/Sensors Based on the Other 2D Materials

Some other 2D materials, such as WS_2_, g-C_3_N_4_, and MnO_2_, have also been engaged to develop advanced sensing systems for electrochemical detection of cancer biomarkers. For example, Toh et al. synthesized WS_2_ sheets on GSC and modified it with Hb and later with glutaraldehyde for the highly sensitive detection of H_2_O_2_ (36 nM) with wide detection range (2–38; 48–1728 µM) [193]. When compared with MoS_2_, MoSe_2_, and WSe_2_, considerably higher performance was observed from the developed biosensor, illustrating the prospective applications of WS_2_. In another report, three-dimensional WS_2_ nanosheet networks were synthesized for monitoring the secreted H_2_O_2_ from living RAW 264.7 macrophage cells and neurons [194]. Using the chemical vapor deposition (CVD) technique, the WS_2_ nanosheet arrays were formed on different substrates of carbon fiber cloths, Ti foil, and nickel foam in a horizontal tube furnace from sulfur and WCl_5_ as precursors. However, in this work, carbon fiber cloth that was made by the conductive carbon fiber network was used due to its low cost, high conductivity, and flexibility, enabling high catalytic administration per geometric area. The fabricated label-free chemosensor represented an extremely low LOD of 2 nM, high selectivity, and potential ability for large-scale production to pave the way for clinical applications. Yang et al. explored the potential ability of WS_2_ for PIK3CA gene (gastric carcinoma biomarker) detection [195]. The WS_2_ nanosheets were dripped on the CPE and later poly(xanthurenic acid) (PXa) was electropolymerized on the surface. Possessing abundant carboxyl groups on the surface of PXa, amine-modified ssDNAs could covalently bind to the surface of the modified electrode. In a label-free fashion using the EIS technique, the developed biosensor could specifically detect the target strand with the LOD of 16 fM.

Duan et al. fabricated a bifunctional aptasensor for PSA detection while using both electrochemical and plasmonic methods on the basis of g-C_3_N_4_ nanosheets and MoS_2_ quantum dots that were decorated with chitosan-stabilized Au nanoparticles [152]. The electrochemical method represented three times higher sensitivity (0.71 pg·mL^−1^) than that of the plasmonic method (0.77 ng·mL^−1^), due to the combination of the three components to enhance the bio-affinity, biocompatibility, and electrochemical activity of the aptasensor. Recently, a photoelectrochemical biosensor was reported for miRNA-396a detection while using MoS_2_/g-C_3_N_4_/black TiO_2_ as the platform and Histostar@AuNPs as the signal amplifier [196]. MoS_2_/g-C_3_N_4_/black TiO_2_ and AuNPs were sequentially modified on ITO substrate to receive probe DNAs. Target hybridization enabled the conjugation of S9.6 antibody and further coupling with Histostar@AuNPs. The 4-chloro-1-naphthol (4-CN) was oxidized by H_2_O_2_ in the presence of HRP, to produce an insoluble product (benzo-4-chlorohexadienone) on the electrode, leading to notable damp in the detected photocurrent. The sandwiched g-C_3_N_4_ served as the electron donor for MoS_2_ and electron accepter for TiO_2_ to give rise to a sensitive detection of 0.13 fM miRNA, demonstrating the significant function of g-C_3_N_4_ in surface modifications.

The application of MnO_2_ in electrochemical cancer diagnostics has been also investigated. For instance, Shu et al. reported a simple and label-free approach for ultra-sensitive detection of H_2_O_2_ released from SP2/0 myeloma cancer cells based on MnO_2_/GCE [197]. Ultra-thin MnO_2_ nanosheets were deposited on GCE while using Nafion. The porous architect and spacious surface area of MnO_2_ provided high electrocatalytic activity toward H_2_O_2_ reduction with the LOD of 5 nM. The constructed platform displayed a good selectivity, sound reproducibility and successful ability to monitor extracellular H_2_O_2_ released from cancer cells, being a good candidate for the development of more practical devices. Very recently, Sha et al. could shorten the response time of electrochemical detection of NO_2_^−^ to less than five seconds based on α-MnO_2_-nanorods hierarchical MoS_2_ microspheres composite [184]. In an optimal condition, a high sensitivity (515.84 µA·mM^−1^·cm^−2^; LOD = 16 µM), pronounce selectivity and reproducibility was achieved within the linear range of 100 to 800 µM. The fast and sensitive performance of the chemosensor can be associated with the heterogeneous interface between two materials α-MnO_2_ nanorods and MoS_2_ to enhance the charge mobility within the structures together with the vast numbers of active sites existed in MoS_2_ edges as well as the higher ratio of metallic (1T) phase to semiconducting (2H) phase in MoS_2_.

## 10. Pros and Cons of Different Detection Systems

Over the past decade, advances in 2D materials-based biotechnology have been accompanied with the development of numerous electrochemical biosensors and sensors. Most of the discussed 2D material-based electrochemical biosensors have shown the advantages of high sensitivity, cost-effectiveness and simplicity to develop/re-develop very accurate detection devices. In addition, some of these sensing platforms have great potential to meet various requirements of POC cancer screening, as they have displayed the ability to efficiently perform tasks in real samples. Nevertheless, there still exits some shortcomings regarding the use of 2D materials in POC cancer screening, as follows:

### 10.1. Type of 2D Material

Beside enormous attention on the 2D material-based biosensors/sensors, however, graphene and its derivatives have been more explored for the application of POC cancer screening, which might be due to their earlier discovery. Due to of the observed exotic features of the reviewed 2D materials and their excellent involvement in enhancing the electrochemical sensing performances, it is highly demanding that the researchers in interdisciplinary areas have more focus on these materials with the aim of fabricating practical diagnostic devices towards reducing the burden of cancer by early detection and accurate treatment.

### 10.2. Reproducibility and Stability

2D material-based electrochemical devices have represented very high sensitivity, however their relatively weak reproducibility is still a challenge to overcome. Identical size distribution and robust surface functionalization are the two factors that affect signal reproducibility. Among them, size-controlled synthesis of graphene and somewhat MoS_2_ have been nearly achieved, although their surface functionalization is still not a straight-forward method. Bi_2_Se_3_ is also suffering from lack of of surface functionalization and biocompatibility. On the other hand, MXene-Ti_3_C_2_ possesses an excellent ability to be functionalized with variety of biomolecules due to the abundance of various functional groups on its surface. However, unfortunately, MXene-Ti_3_C_2_ is seen to be unstable in the anodic potential window to hamper its applications in electrochemical biosensing. Moreover, the production of monodispersed and homogenous MXene-Ti_3_C_2_, WS_2_, MnO_2_ and g-C_3_N_4_ nanosheets is yet to be achieved. Even though the combination of two or three 2D-materials has been shown to elevate some of these challenges, comprehensive efforts are needed to address the matter.

### 10.3. Type of Biomarker

According to the reviewed articles, in the direction of POC cancer screening, the maximum attention was toward protein biomarkers followed by small molecules and later the nucleic acids. This might be due to (**i**) the ability of protein-recognition elements to rapidly identify and conjugate with the target protein biomarkers, which is quite slower in case of nucleic acids, and (ii) their higher involvement in the accurate detection of cancers when compared with the small molecules (NO and H_2_O_2_). However, due to the fact that nucleic acid biomarker detection systems offer lower price and higher selectivity (sequence-specific mechanisms) alongside the ease of multiplexing and providing very useful information about the early development stage of tumors, there is a real demand to have more research concentration on this category for POC cancer screening.

### 10.4. Multiplexing

As a one of the best cancer biomarkers, PSA can only provide 70% predictive measure [198], which truly explains the importance of multiplexing in the accurate and reliable diagnosis of cancer. Various 2D material-based electrochemical platforms have been reported, however they are mainly capable of only one biomarker detection. Accordingly, it is necessary to fabricate a single platform for multiple biomarker detection or exploring other alternative mechanisms.

### 10.5. Sample Preparation and Clinical Validation

Using a proper sample purification step and very specific biorecognition elements would be desirable due to the high possibility of non-specific bindings and biofouling effects in electrochemical biosensors. Although, MXene-Ti_3_C_2_ has shown a great capability towards elimination of non-specific adsorptions, making it very interesting material in electrochemical sensing applications. Moreover, most of the developed 2D material-based electrochemical devices have shown very high performances toward model biomarkers, which means that only few biosensors/sensors have performed in clinical samples. This might be either due to the lack of collaboration between the research groups or limited financial supports.

### 10.6. POC Devices

Current commercially-available electrochemical devices (e.g., ELISHA (http://www.immunosensors.com/), LumiraDx (http://www.poct.co.uk/index.cfm), etc.) are not based on 2D materials or designed for cancer biomarker detection. Moreover, none of them can perfectly satisfy the specifications of an ideal POC cancer screening device to be (**i**) simple, (ii) affordable, (iii) multiplex, (iv) portable, (**v**) fully-automated, and (vi) require low energy supply. Even though, recently, Ainla et al. reported a prototype electrochemical POC device as an open-source universal wireless electrochemical detector (UWED) (Figure 1e), being able to transfer data to smartphone via Bluetooth and further analysis and cloud storage, it still requires more studies towards integration with 2D materials for multiplex and accurate detection of various cancer biomarkers in clinical samples [14]. However, the commercial implementation of electrochemical POC cancer screening relies on cost reduction and large-scale production, which gives UWED a suitable case to study.

## 11. Concluding Remarks and Future Perspectives

An ideal POC cancer screening device should represent high accuracy and specificity, short assay process, low price, and accessibility to people living in the downside of advantage. Intrinsic sensitivity, pace, and cost-effectiveness of the electrochemical sensing devices has continued to be a driving force for the fabrication of POC cancer screening devices. In this review, substantial advances in the fabrication of various 2D material-based electrochemical biosensors/sensors was summarized for the detection of cancer biomarkers in the direction to POC screening. Advances in the application of 2D materials in the development of electrochemical cancer biomarker detection was considered, having a particular focus on POC cancer screening. Graphene and its derivatives, MoS_2_ in the form of nanosheets and nanoparticles, TI Bi_2_Se_3_, MXene-Ti_3_C_2_ nanoflakes, as well as WS_2_, MnO_2_, and g-C_3_N_4_ nanosheets were reviewed and compared. The function of each material into the elevating the sensor performances were highlighted. Thought-provoking outcomes can be extracted from this review, such as the trends in the development of 2D material-based electrochemical sensing devices and implementing different methods to improve their efficiency. The integration of nanostructures with these 2D materials could provide synergetic impacts on the electrocatalytic activity of constructed platforms by increasing the surface area as well as the charge mobility throughout the structure. Moreover, the combination of different 2D materials resulted in the amplification of the electrochemical signals, together with improvement in their surface functionalizations.

Towards POC cancer screening, many research efforts have been made, such as graphene-based SPCE for ctDNA and miRNA quantification, multiplex protein biomarker quantification using graphene-modified µPAD, sandwich assay based on graphene nanosheets on microfluidic device for protein biomarker detection, aptamer/graphene-based µPAD for dual protein biomarker quantification, GO-modified electrode for H_2_O_2_ screening based on µPAD, MoS_2_-based sandwich assay for multiplex profiling of miRNA using microfluidic chip, protein biomarker detection using MoS_2_-based µPAD, and non-enzymatic and enzymatic H_2_O_2_ quantification based on MoS_2_-modified SPCE and PCB, respectively. These examples can provide good insights of the practical applications and promising potentials of 2D materials to be actively engaged in the development of competent electrochemical POC cancer screening devices.

Additional researches and developments are highly required to construct reliable and fully-automated POC cancer screening devices besides the observed remarkable progress in the fabrication of different electrochemical biosensors and sensors for cancer biomarker detection based on 2D materials. Up until now, most of the studies have been concentrated on the methodology rather than the device manufacturing. As such, more endeavors need to be carried out with intensive help from different fields of microelectronics (designing miniaturized electronic chips), microfluidics (implementing automation), device manufacturing (providing portable devices), computer programing (constructing mobile Apps), chemical engineering (designing different sensing architects), chemistry (understanding the chemical reactions), physics (comprehending the charge transfer mechanisms), and biology (understanding the biological pathways and applying clinical validations) to develop qualified electrochemical POC cancer screening devices for precise testing and analysis within a few minutes at the patient bedside.

## Figures and Tables

**Table 1:** Graphene-based electrochemical genosensors for nucleic acid detection. The underlined terms are the bioreceptors.

Electrode Architect	Target	Detection Method	Label	LOD	Linear Range	Real Sample	Device	Ref.
**Ab2-HRP/Ab1/ssDNA/o-MWCNT/RGO/GSP**	miR-141 and miR-29b-1	DPV	anti-HRP	10 fM	10 fM to 1 nM	No	No	[73]
OB/ssDNA/GO/GNR/GCE	miR-155	DPV	Oracet Blue (OB))	0.6 fM	2.0 fM to 8.0 pM	Human plasma samples	No	[74]
ssDNA/SA/Au-PtBNPs/CGO/FTO	miRNA-21	DPV	No	1 fM	1 fM to 1 μM	Spiked human serum	No	[78]
SA-ALP/ssDNA-GO-AuNPs/ssDNA/AuNPs/MgO/GCE	miRNA-21	DPV	SA-ALP	0.05 pM	0.0001 to 100 pM	Human serum samples	No	[75]
ssDNA/NFG/AgNPs//FTO	miRNA-21	DPV	No	0.2 fM	10 fM to 10 μM	Human blood samples	No	[76]
SA-dynabeads /ssRNA/GO-IO/SPCE	miRNA-21	CA	SA-dynabeads	1.0 fM	1.0 fM to 1.0 nM	No	SPCE	[77]
N-doped-Multiple Graphene Agarose /AuNPs/GCE	ctDNA	DPV	No	3.9 ng mL^−22^	1.0 × 10^−21^ to 1.0 × 10^−16^ ng mL^−1^	Human serum samples	No	[79]
SA-dynabeads /ssRNA/GO-NPFe_2_O_3_/SPCE	ctNAs- FGFR2:FAM76A	CA	SA dynabeads	1 fM	1.0 fM to 1.0 nM	Human plasma samples	No	[80]
Au@ SCX8- Au@SCX8-rGO-TB-LP/AP/ssRNA/Au@Fe_3_O_4_/SPCE	mRNA (CCND2-S and CCND2-L)	DPV	Au@SCX8-RGO-TB-LP	CCND2-S: 0.176 aM; CCND2-L: 9.5 aM	CCND2-S: 10^−18^ to 10^−11^ and CCND2-L: 10^−17^ to 10^−11^ M	Lung cancer cells	SPCE	[71]
MB/ssDNA/3D GF/AgNPs	CYFRA21-1 DNA	DPV	MB (MethylBlue)	1.0 × 10^−14^ M	1.0 × 10^−14^ to 1.0 × 10^−7^ M	Lung cancer cells	No	[81]
Ni-OTC NPs/ssDNA-OMC/rGO/PGE	EGFR exon 21point mutation	DPV	Ni-OTC NPs	120 nM	0.1 μM to 3 μM	No	No	[82]
SA-HRP /ssDNA/rGO-CMC/SPCE	TP53 Gene	A	SA-HRP	sccp53: 3.4 nM, lcpp53: 2.9 nM	0.01 to 0.1 μM	Total human serum and saliva	SPCE	[70]
ssDNA reporter-AuNP/ssDNA /Gr/ITO	BRCA1 gene	CA	DNA-AuNP	1 fM	1 fM to 1 nM	No	No	[69]

**Table 2:** Graphene-based electrochemical biosensors for protein cancer biomarker detection. The underlined terms are the bioreceptors.

Electrode Architect	Target	Detection Method	Label	LOD	Linear Range	Real Sample	Device	Ref.
rGO-FA/Au	Folic acid protein (FP)	CV/DPV	No	1 pM	1–200 pM	No	No	[85]
Ab/PEDOT:PSS/rGO/Whatman filter paper electrode	CEA	EIS/ CA	No	25.8 µA ng^−1^ mL cm^−2^	1–8 ng·mL^−1^	Human serum samples	No	[88]
Ab/AuNPs/Thi/rGO/Pure cellulose paper electrode	CA125	DPV	No	0.01 U mL^−1^	0.1 U·mL^−1^–200 U·mL^−1^	Human serum samples	No	[91]
Apt/AuNPs/rGO/THI/Cellulose paper electrode	PSA	CV/DPV	No	10 pg mL^−1^	0.05 to 200 ng·mL^−1^	Human serum samples	Paper microfluidic device	[92]
MB/Apt/rGO-Chit/GCE	HER2	DPV	MB	0.22 ng mL^−1^	2–75 ng·mL^−1^	Human serum samples	No	[87]
MWCNT-COOH /PEDOT/Graphene/Cellulose paper electrode	OS- 8-OHdG	CV/DPV	No	14.4 ng mL^−1^	50–1000 ng·mL^−1^	Human serum samples	Paper-Based Sensing Device	[93]
Ab2/PdPt nanocages/MWCNT/Ab1/NH_2_-GS/GCE	CEA	A	PdPt nanocages	0.2 pg mL^−1^	0.001–20 ng·mL^−1^	Human serum samples	No	[94]
**Ab**-HRP/pDA/3D-Graphene	CEA	DPV	HRP	90 pg mL^−1^	0.1–750.0 ng·mL^−1^	Human serum samples	No	[95]
SA-HCR/Ab2/Ab1/ Graphene–Au/GCE	AFP, CEA, CA125, PSA	DPV	SA-HCR	62, 48, 77 and 60 fg mL^−1^	0.2 to 800 pg·mL^−1^ for AFP, 0.2 to 600 pg·mL^−1^ for CEA, 0.2 to 1000 pg·mL^−1^ for CA125, 0.2 to 800 pg·mL^−1^ for PSA	Human serum samples	No	[96]
Ab/ZrO_2_–rGO/ITO	CYFRA-21-1	DPV	No	0.122 ng mL^−1^	2–22 ng mL^−1^	Human saliva samples	No	[97]
HRP/Ab2/Ab1-Fe_3_O_4_@GO/Carbon electrode	PSA and PSMA	A	HRP	15 fg·mL^−1^ for PSA and 4.8 fg·mL^−1^ for PSMA	61 fg·mL^−1^ - 3.9 pg·mL^−1^ for PSA and9.8–624 pg·mL^−1^ for PSMA	Human serum samples	Microfluidic immunoarray device	[89]
Ab/TB/AuNPs/Fe_3_O_4_-rGO/GCE	AFP	A	No	2.7 fg·mL^−1^	1.0 × 10^−5^–10 ng·mL^−1^	No	No	[98]
Ab/CysA-Au NPs/Ag-rGO/Photo paper	CA153	CA	No	15 U·mL^−1^	15–125 U·mL^−1^	Human plasma samples	μPAD	[99]
Ab2-porous-hollowed-Ag-Au/Ab1/Graphene/SPCE	PSA	DPV	Metal-ion-loaded AuNP	0.13 pg·mL^−1^	0 - 80 ng·mL^−1^	Human serum samples	SPCE	[100]
Apt/NH_2_- Graphene-THI-AuNPs and PB-PEDOT- AuNPs/SPE	CEA, NSE	DPV	No	2 pg·mL^−1^ for CEA and 10 pg·mL^−1^ for NSE	0.01–500 ng·mL^−1^	Human serum samples	Microfluidic paper-based device	[90]
HRP-Ab2-SiO_2_/Ab1/GO/chitosan/Pure cellulose paper	AFP, CEA, CA125, CA153	DPV	HRP	0.001 ng·mL^−1^ for AFP, 0.005 ng·mL^−1^ for CEA, 0.001 ng·mL^−1^ for CA125, and 0.005 ng·mL^−1^ for CA153	0.001−100 ng·mL^−1^ for AFP,0.005−100 ng·mL^−1^ for CEA, 0.001−100 ng·mL^−1^ for CA125, and 0.005−100 ng·mL^−1^ for CA153	Human serum samples	μPAD	[84]
FeONPs and CoZnFeONPs-/Ab2/Ab1/GO/Carbon-based electrode	CYFRA 21-1	A	No	0.3 fg·mL^−1^ for FeONPs and 0.19 fg·mL^−1^ for CoZnFeONs	3.9 to 500 fg·mL^−1^ for FeONPs and 3.9 to 1000 fg·mL^−1^ for CoZnFeONs	Human serum samples	μIDs	[101]

**Table 3:** Reported literature for graphene-based chemosensors for electrochemical detection of NO and H_2_O_2_.

Electrode Architect	Target	Detection Method	LOD	Linear Range	Real Sample	Device	Ref.
MNPs@Y-1,4-NDC-MOF/ERGO/GCE (M = Ag, Cu)	H_2_O_2_	A	0.18 μM	4 to 11,000 μM	Living cells	No	[120]
NSG/GCE	H_2_O_2_	A	1 μM	4 to 50 μM	Living cells	No	[110]
AuNPs-ctDNA-NGS/SPCE	NO	A	0.8 nM	2 to 500 nM	Living cells	SPCE	[121]
Au-Pd/rGO/GCE	H_2_O_2_	A	0.004 μM	0.005 to 3500 μM	Human breast cancer cells	No	[122]
rGo/AuNP/Paper electrode	H_2_O_2_	CV	15 μM	8.53 to 17.35 mM	No	No	[123]
Au@PtNP/GO nanozymes/ITO	H_2_O_2_	CA	1.62 μM	0.001 to 3 mM	Human urine samples	Electrochemical microfluidic devices	[119]
Ag-PdNPs/rGO/GCE	H_2_O_2_	CV/A	1.1 μM	0.005 to 14.65 mM	Human urine samples	No	[124]
Pt/Graphene-CNT/Paper electrode	H_2_O_2_	A	10 nM	0.1 To 25 μM	Living cells	No	[118]
AuNPs/N-GQDs/GCE	H_2_O_2_	CV/A	0.12 μM	0.25 to 13327 μM	Human serum samples and living cells	No	[125]
N-Graphene/GCE	H_2_O_2_	A	0.05 μM	0.5 mM to 1.2 mM	Living cells	No	[103]
(Graphene-AP-laminin)_10_/ITO	H_2_O_2_	A	0.1 μM	Up to 100 μM	Living cells	No	[108]

**Table 4:** Reported studies of molybdenum disulfide (MoS_2_)-based electrochemical biosensors for nucleic acid cancer biomarker detection. The underlined terms are the bioreceptors.

Electrode Architect	Target	Detection Method	Label	LOD	Linear Range	Real Sample	Fabricated Device	Ref.
ssDNA/nanoMoS_2_/CPE	ssDNA	DPV	No	1.9 × 10^−17^ M	10^−16^–10^−10^ M	No	No	[136]
ssDNA/MoS_2_-graphene/CGE	ctDNA	DPV	No	10^−17^ M	10^−16^–10^−13^ M	No	No	[131]
ssDNA/MoS_2_-polyaniline/Pt	ssDNA	DPV	No	10^−15^ M	10^−15^–10^−6^ M	Human serum	No	[137]
ssDNA/PXA-MoS_2_/CPE	ctDNA	EIS	No	1.8 × 10^−17^ M	10^−16^–10^−10^ M	No	No	[138]
ssDNA/PANI-MoS_2_/ITO	ssDNA	EIS	No	3 × 10^−18^ M	10^−6^–10^−17^ M	CML positive patient samples	No	[139]
ssDNA/PIn_6_COOH-MoS_2_/CPE	ctDNA (PIK3CA gene)	EIS	No	1.5 × 10^−17^ M	10^−16^ M–10^−1^ M	No	No	[140]
DNA_2_/DNA_1_/AuNPs@MoS_2_/GCE	miRNA-21	DPV; EIS	No	0.78 fM (DPV); 0.45 fM (EIS)	10 fM–1 nM	Human serum	No	[132]
ssDNA/AuNPs@MoS_2_-Thi/GCE	miRNA-21	DPV	No	0.26 pM	1.0 pM–10 nM	Human serum	No	[133]
ssDNA/AuNPS@hollow MoS_2_ microcubes/GCE	miRNA-21	DPV	No	0.086 fM	0.0001–100 pM	Human breast cancer serum	No	[134]
H_2_/H_1_/cDNA/AuNPs@Carbon sphere-MoS_2_/GCE	miRNA-21	DPV	HRP	1.6 × 10^−17^ M	0.0001–100 pM	Human serum	No	[135]
Strp-MMBs/ssDNA/MoS_2_-CuFe_2_O_4_	multiplexed miRNA	SWV	FcSH	0.48 pM	1 pM–1.5 nM	Human serum; positive clinical samples - blood and fecal samples	Microfluidic biosensor	[141]
ssRNA/AuNPs@MoS_2_-Ti_3_C_2_/GCE	miRNA-182	DPV	No	0.43 fM	1 fM–0.1 nM	Human serum	No	[142]
PtCuMOF-DNA_3/4_/DNA_1/2_/MoS_2_/AuNPs/AgNW	miRNA-141; miRNA-21	SWV	G4/Hemin	0.1 fM	1 fM–1 nM	Human serum	Paper-based biosensor	[143]

**Table 5:** MoS_2_-based electrochemical biosensors for protein cancer biomarkers quantification. The underlined terms are the bioreceptors.

Electrode Architect	Target	Detection Method	Label	LOD	Linear Range	Real Sample	Device	Ref.
Fc-AuNPs-Apt2/Apt1/AuNPs/MoS_2_/carbon aerogel/GCE	PDGF-BB	DPV	FcSH	0.3pM	0.001–10nM	Human serum	No	[144]
Anti-CEA/AuNPs-Thi-MoS_2_/GCE	CEA	SWV	No	0.52 pg/mL	1 pg/mL–10 ng/mL	Human serum	No	[147]
Ab_2_/Ab_1_/MoS_2_-Au/GCE	CEA	DPV	Ag nanosphere	0.27 pg/mL	1 pg/mL–50 ng/mL	Human serum	No	[148]
Hemin/PdNPs/PDDA-G-MoS_2_/TBA-NH_2_/GCE	Thrombin	DPV	G4/Hemin	0.062 pM	0.0001–40 nM	Human blood serum	No	[145]
GOx-AuNF@Ab_2_/Ab_1_/MoS_2_-Au/Paper	CA125	DPV	GOx	0.38 pg/mL	0.001–50 ng/mL	Human serum	Microfluidic paper-based	[150]
HRP-DNA/Ab_2_/Ab_1_/AuNPs@hollow MoS_2_ microbox/GCE	AFP	DPV	HRP	2.0 fg/mL	0.00005–75 ng/mL	Human serum	No	[151]
Apt/MoS_2_QDs@g-C_3_N_4_@CSAuNPs	PSA	EIS	No	0.72 ng/mL	1.0–250 ng/mL	Human serum	No	[152]
Anti-PSA/MoS_2_/Au	PSA	EIS	No	0.001 ng/mL	0.001–200 ng/mL	Human serum	No	[153]
Anti-PSA-Ag/MoS_2_@Fe_3_O_4_/AgNPs/GCE	CEA	DPV	No	0.03 pg/mL	0.0001–20 ng/mL	Human serum	No	[154]
Anti-CEA/MoS_2_-PBNCs/GCE	CEA	DPV	No	0.54 pg/mL	0.005–10 ng/mL	Human serum	No	[148]
Anti-CEA/ Ag/MoS_2_/rGO/GCE	CEA	A	No	1.6 fg/mL	0.01 pg/mL–100 ng/mL	Human serum	No	[155]
HRP-Ab_2_/Ab_1_/MoS_2_-AuNPs/GCE	CEA	DPV	HRP	1.2 fg/mL	10 fg/mL^–1^ ng/mL	Human serum	No	[149]

**Table 6:** Reported studies for MoS2-based electrochemical detection of H_2_O_2_ and NO. The underlined terms are the bioreceptors.

Electrode Architect	Target	Detection Method	LOD	Linear Range	Real Sample	Device	Ref.
Hb/AuNPs@MoS_2_/GCE	H_2_O_2_; NO	CV	H_2_O_2_: 4 µM; NO: 5 µM	H_2_O_2_: 10–300 µM; NO: 10–1100 µM	No	No	[171]
IgG-HRP/MoS_2_-Au/PCB	H_2_O_2_	CV	No	0–20 ng/mL	No	No	[172]
Laminin/Au−Pd−Pt/MoS_2_/SPCE	H_2_O_2_	CV	0.3 nM	1–100 nM	MCF-7 cancer cells	SPCE	[165]
Nafion/Hb/MoS_2_-rGO/GCE	H_2_O_2_	CV	25 nM	0.1–250 µM	No	No	[156]
PtW/MoS_2_/GCE	H_2_O_2_	A	No	Up to 5 nM	Breast cancer 4T1 cells	No	[162]
Nafion/Mb/MoS_2_ -graphene/ Nafion/GCE	H_2_O_2_; NaNO_2_	CV	H_2_O_2_: 1.25 µM; NaNO_2_: 0.125 mM	H_2_O_2_: 6.25–225 µM; NaNO_2_: 1.25–12.5 mM	H_2_O_2_: Milk sample	No	[172]
CuNFs/MoS_2_/GCE	H_2_O_2_	A	0.021 µM	0.04–1.88 µM	Tap water	No	[157]
MoS_2_-PtNP/GCE	H_2_O_2_	A	0.345 µM	0.02–4.72 mM	No	No	[174]
MoS_2_-PBNCs/GCE	H_2_O_2_	A	4.1 nM	0.01–300 µM	No	No	[148]
Mb/GO@MoS_2_/Au	H_2_O_2_	A	20 nM	-	No	No	[175]
Catalase/MoS_2_-Au/chitosan/GCE	H_2_O_2_	A	10^−7^ M	5 × 10^−7^–2 × 10^−4^ M	SP2/0 cells	No	[164]
Au-Pd/MoS_2_/GCE	H_2_O_2_	DPV	0.16 µM	0.8 µM–10 mM	No	No	[158]
MoS_2_/PtNPs/Au/AN	H_2_O_2_	DPV; A	0.686 µM	1–100 µM	HeLa cells	No	[176]
**PB**/MoS_2_-rGO/GCE	H_2_O_2_	A	0.14 µM	0.0003–1.15 mM	Tap & river water	No	[160]
O-MoS_2_/graphene/GCE	H_2_O_2_	A	0.12 µM	0.25–16 mM	Water	No	[159]
Pt-Pd/MoS_2_/GCE	H_2_O_2_	A	3.4 µM	10 to 80 µM	No	No	[177]
MoS_2_-OCu/GCE	H_2_O_2_	A	0.0767 µM	0.085–38.0 mM	No	No	[178]
MoS2-ICPC/GCE	H_2_O_2_	A	11.8 µM	20–300 µM	No	No	[179]
3D RGO-MoS_2_QDs/GCE	H_2_O_2_	A	1.90 µM	0.01–5.57 mM	No	No	[161]
MoS_2_/C_N_ NWs/GCE	H_2_O_2_	A	0.73 µM	2–500 µM	Epithelial cells (A549 cells)	No	[163]
Pt/MoS_2_/Ti	H_2_O_2_	A	0.87 µM	10–160 µM	No	No	[180]
Fe_2_O_3_@MoS_2_/Nafion/GCE	NO_2_^−^	A	1.0 µM	2.0–6730 µM	Drinking water; River water	No	[166]
TOSC-MoS_2_/GCE	NO_2_^−^	A	2 µM	6–3140, 3140–4200 µM	Drinking water; River water	No	[167]
Fe_3_O_4_/MoS_2_/GCE	NO_2_^−^	A	0.5 µM	1–2630 µM	No	No	[181]
Mb/GO/Amine-modified MoS_2_/Au	NO	A	3.6 nM	-	No	No	[173]
AuNPs@MoS_2_-NSs/GCE	NO_2_^−^	DPV	0.5 µmol/L	5.0–260.0 µmol/L	Human serum	No	[168]
Ag/HNT/MoS_2_/CPE	NO_2_^−^	A	0.7 µM	2–425 µM	Tap water and aqueduct water	No	[182]
rGO-MoS_2_/GCE	NO_2_^−^	A	0.17 µM	0.2–4800 µM	Tap water	No	[183]
AuNPs/MoS_2_/GCE	NO_2_^−^	A	1.67 µM	5.0 µM–27.8 mM	Tap water	No	[169]
α-MnO_2_-MoS_2_/GCE	NO_2_^−^	A	16 µM	100–800 µM	Drinking water	No	[184]
PrFeO_3_-MoS_2_/GCE	NO_2_^−^	CV	1.67 µM	0.005–3 mM	Tap water; Local river water and waste water	No	[185]
